# Mechanosensitive Ion Channel PIEZO1 Suppresses BMP2‐Induced Ossification of the Annulus Fibrosus Cells

**DOI:** 10.1002/jsp2.70168

**Published:** 2026-03-03

**Authors:** Hisakazu Shitozawa, Ryo Nakamichi, Aki Yoshida, Masataka Ueda, Taichi Saito, Koji Uotani, Yoshiaki Oda, Ryo Takatori, Kazutaka Yamashita, Toshifumi Ozaki

**Affiliations:** ^1^ Department of Orthopaedic Surgery, Science of Functional Recovery and Reconstruction Okayama University Graduate School of Medicine, Dentistry and Pharmaceutical Sciences Okayama Japan; ^2^ Department of Orthopaedic Surgery Okayama University Graduate School Medicine, Dentistry, and Pharmaceutical Sciences Okayama Japan; ^3^ Department of Orthopaedic Surgery Okayama University Hospital Okayama Japan; ^4^ Department of Orthopaedic Surgery, Faculty of Medicine, Dentistry and Pharmaceutical Sciences Okayama University Okayama Japan

**Keywords:** annulus fibrosus, calcification, ossification, PIEZO1

## Abstract

**Objective:**

Major cause of low‐back pain is intervertebral disc degeneration (IVDD), with mechanical stress playing a crucial role in its progression. A mechanosensitive ion channel, PIEZO1, is involved in various musculoskeletal tissues, but its role in the annulus fibrosus (AF) remains unclear. This study aimed to elucidate the function of PIEZO1 in AF cells under mechanical stimulation.

**Methods:**

Primary rat AF cells were subjected to cyclic tensile strain (CTS) at low (2%) and high (12%) strain levels to investigate strain‐dependent effects on osteogenic gene expression. We evaluated the effects of *Piezo1*, *Piezo2*, and *Trpv4* knockdown by RNA interference to identify the upstream mechanotransducer. Furthermore, PIEZO1 was activated using the agonist Yoda1, followed by RNA‐sequencing analysis and evaluation of its effects on BMP2‐induced osteogenesis in rat AF cells. We also examined the effects of Yoda1 in primary human AF cells.

**Results:**

Low‐strain CTS significantly suppressed osteogenic marker expression, which was not observed with high strain. *Piezo1* knockdown reversed this suppression, whereas *Piezo2* and *Trpv4* had no effect. Piezo1 activation by Yoda1 produced similar anti‐osteogenic effects in both rat and human AF cells. RNA sequencing revealed the enrichment of ossification and calcineurin signaling pathways in rat cells. Furthermore, Piezo1 activation inhibited BMP2‐induced osteogenesis and nuclear translocation of p‐Smad1/5/9.

**Conclusions:**

Piezo1 maintains AF cell homeostasis under mechanical stress by suppressing osteogenic changes via calcineurin‐mediated inhibition of BMP signaling, which may represent a novel therapeutic target for IVDD.

## Graphical abstract

This study aimed to elucidate the function of PIEZO1in AF cells under mechanical stimulation. Low‐magnitude cyclictensile strain (CTS, 2%) suppressed osteogenic marker expression. Pharmacological activation of PIEZO1 using Yoda1 mimics the effects oflow‐strain CTS. Transcriptomic analysis revealed enrichment of ossification‐relatedand calcineurin signaling pathways. PIEZO1 activation inhibits BMP2‐inducedosteogenesis and nuclear translocation of p‐Smad1/5/9, thereby maintaining AFcell homeostasis under physiological mechanical stress.

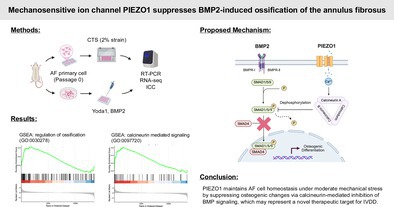

## 1 Introduction

Low‐back pain (LBP) is a leading cause of disability that affects 12%–35% of individuals in daily life [1]. Approximately 40% of LBP cases are attributed to intervertebral disc (IVD)‐related issues, particularly intervertebral disc degeneration (IVDD) [2].

The IVD consists of the nucleus pulposus (NP) and annulus fibrosus (AF) [3]. The NP contains randomly arranged type II collagen fibers and radially oriented elastin fibers, embedded within the aggrecan‐rich gel matrix. Conversely, the AF consists of 15–25 concentric lamellae wherein collagen fibers are aligned parallel within each lamella. The AF is further subdivided into inner and outer regions composed of type II and I collagen fibers, respectively [2, 4]. At the cellular level, NP cells are chondrocyte‐like and inner AF cells also exhibit a rounded, chondrocyte‐like morphology, in contrast to the spindle‐shaped, fibroblast‐like morphology of outer AF cells. Inner AF cells express type II collagen (COL2A1) and fibromodulin [4, 5, 6], whereas outer AF cells predominantly express type I collagen (COL1A1), type V collagen, and secreted frizzled‐related protein 2, a modulator of Wnt signaling [7].

Typically, IVDD begins in late adolescence and progresses with aging [8]. This degenerative process affects the disc at the molecular, cellular, and tissue levels, altering morphology and physiological function. Early histological signs of IVDD include loss of water content and depletion of proteoglycan in the NP, which leads to reduced elasticity. These changes disrupt the lamellar structure and cause AF tearing, facilitating aberrant vascular and nerve ingrowth into the disc. The vascular invasion from the cartilaginous endplate and anterior AF can promote ossification and calcification within the disc. Ossification is often associated with irregular granular deposits accompanied by rupture or necrosis in the AF [2, 8, 9, 10]. Chanchairujira et al. reported that 80% of cadaveric specimens exhibited intervertebral disc calcification, with 63% occurring in the AF. Furthermore, disc calcification was significantly correlated with age and intervertebral disc space narrowing [11]. Consistent with these findings, human AF cells derived from degenerated IVDs exhibit increased expression of osteogenic genes, such as Runt‐related transcription factor 2 (RUNX2), alkaline phosphatase (ALP), and osteocalcin (OCN) [12].

Bone morphogenetic protein‐2 (BMP2) is a well‐characterized inducer of osteogenesis in AF cells, particularly under mechanobiological stimulation. Mechanical loading has been shown to strongly activate BMP signaling in AF tissue. For example, high cyclic tensile strain (CTS) upregulates BMP‐2/6 heterodimer expression and activates p38 MAPK and Smad1/5/8 signaling in human AF cells, resulting in increased expression of RUNX2 and osterix (OSX) and promotion of osteogenic differentiation [13]. Human AF cells cultured under osteogenic conditions exhibit increased expression of BMP2 and other osteogenic markers, such as osteopontin and RUNX2 [14]. BMP2 and its receptors are also expressed in vivo in the AF and CEP [15], and degenerated human discs show marked upregulation of BMP2 and phosphorylated SMAD1/5/8 that correlates with Pfirrmann grade [16]. AF cells exhibit robust BMP2‐dependent osteogenesis in vitro, and BMP2 administration has been shown to induce AF calcification in ex vivo IVD cultures [17]. Collectively, these findings support BMP2 as a physiologically and mechano‐biologically relevant inducer of osteogenic differentiation in AF cells.

IVDD is a multifactorial disorder influenced by genetic predisposition, aging, lifestyle (such as obesity, smoking, and depression), and mechanical loading [18], with excessive mechanical stress inducing microscopic damage to the IVD, leading to degenerative changes and contributing to LBP [19]. Conversely, moderate exercise therapy can effectively treat chronic LBP by improving function and enhancing work capacity [20]. The mechanical stress on AF cells exerts different effects at the cellular level, depending on the stress intensity. High‐intensity mechanical loading promotes apoptosis in AF cells [21], increases prostaglandin E2 synthesis and inflammatory mediator production [22], and reduces anabolic activity, impairing disc homeostasis [23]. By contrast, low‐ to moderate‐intensity mechanical loading suppresses catabolic gene expression and promotes anabolic processes under inflammatory conditions [24, 25]. Importantly, because the annulus fibrosus is normally a fibrous tissue, osteogenic differentiation must be actively restrained under physiological conditions. In this context, appropriate mechanical stress not only avoids osteogenic activation but may actively suppress osteogenic transcriptional programs in AF cells, thereby maintaining tissue identity.

Mechanosensitive ion channels play a crucial role in mechano‐transduction by converting mechanical energy into electrical or biochemical signals [26]. Several such channels have been identified, including degenerins/acid‐sensitive channels (DEG/ENaC), TREK/TRAAK channels, transient receptor potential channels, PIEZO channel, and TMEM16 superfamily [27]. Among these, PIEZO1, a mechanical stress‐responsive calcium channel receptor, functions across various tissues, including the musculoskeletal system [28]. In the NP, PIEZO1 mediates inflammation and apoptosis, contributing to the onset and progression of IVDD [29, 30]. In contrast, AF cells share molecular features with fibrous connective tissues such as tendons and ligaments, which express the transcription factors Mohawk (Mkx), Scleraxis (Scx), and tenomodulin [31, 32, 33]. These factors are largely absent in NP cells. Notably, PIEZO1 activation has been reported to enhance tendon function [34]. Given the structural and functional similarities between tendon tissue and the AF, PIEZO1 may exert distinct roles in the AF compared with the NP. In the AF, PIEZO1 senses excessive mechanical loading and induces apoptosis via the Calpain1/BAX/Caspase3 pathway, thereby accelerating IVDD [35]. Importantly, calcium influx through PIEZO1 has been reported to be intensity dependent [36], suggesting that the magnitude of Ca^2+^ entry may influence downstream biological effects. Likewise, the cellular response of AF cells to mechanical stimulation is highly dependent on strain intensity. Under physiological conditions, the average strain on AF is approximately ≤ 6% [37]. Zhang et al. reported that moderate physiological loading promotes anabolic processes in AF cells, increasing Col1a1, Col2a1, and ACAN expression [25]. Such loading also downregulates caveolin‐1, resulting in suppression of NF‐κB signaling and reduced pro‐inflammatory cytokines expression, such as COX‐2, IL‐1β, IL‐6, and TNF‐α, thereby exerting anti‐inflammatory effects [25]. Collectively, these findings indicate that excessive mechanical loading accelerates AF degeneration, whereas low‐ to moderate‐intensity loading contributes to homeostasis maintenance, potentially offering protective effects against disc degeneration.

Given these considerations, this study aimed to identify the mechanoreceptors involved in AF homeostasis under moderate mechanical stimulation and elucidate their functional roles through in vitro experiments. Our findings demonstrate that low to moderate mechanical stimulation induces an anti‐ossification effect in AF cells and that PIEZO1 plays a key role in this process.

## 2 Materials and Methods

### 2.1 Rat AF Primary Cell Isolation

Primary AF cells were isolated from 10‐week‐old male Wistar rats (Jackson Laboratory Japan, Kanagawa, Japan). The Institutional Animal Care and Use Committee of Okayama University approved all animal experiments (protocol no. OKU‐2023501).

A total of 72 rats were used for primary cell isolation. Animals were euthanized by CO_2_ inhalation, and the outer AF from the L1–S1 levels was carefully extracted and enzymatically digested to obtain primary cells. Detailed steps for tissue processing, enzymatic digestion, and cell culture conditions are provided in the Supporting Informations and Methods S1. In all experiments, only Passage 0 cells from at least three independent isolations performed at different time points were used.

### 2.2 Human AF Primary Cell Isolation

Human AF cells were isolated from intervertebral discs (IVDs) obtained from two distinct patient groups. In the first group, nondegenerated IVD tissues (Pfirrmann grade 1) were collected from adolescent patients (mean age, 16.0 years) undergoing corrective surgery for adolescent idiopathic scoliosis (AIS). In the second group, severely degenerated IVD tissues (Pfirrmann grade 5) were obtained from older adults (mean age, 75.3 years) undergoing lumbar surgeries, including lateral lumbar interbody fusion or corpectomy, at levels L1–L5. Demographic information for both patient groups is summarized in Supplementary Table S1. The Ethics Committee of Okayama University approved all procedures (approval no. 2212‐021), and all patients provided informed consent preoperatively. Detailed enzymatic digestion steps are provided in the Supporting Informations and Methods S1. We used Passage 1–2 cells from at least three independent patient‐derived cell lines for all experiments.

### 2.3 Cyclic Tensile Strain

To enhance cell adhesion, flexible stretch chambers (2 × 2 cm) (Menicon Life Sciences, Nagoya, Japan, Cat. #SC4Dea) made of polydimethylsiloxane (PDMS) were coated with collagen type I (Corning, NY, Cat. #354236). Cells were seeded at 8.0 × 10^4^ and cultured at 37°C in a 5% CO_2_ incubator for 48 h. Subsequently, cells were cultured under low‐serum conditions (2% FBS) for 12 h before being subjected to CTS using the ShellPa Pro (Menicon Life Sciences). We applied two different mechanical strains: low‐ (strain: 2%, frequency: 1 Hz, duration: 12 h) and high‐strain stimulation (strain: 12%, frequency: 1 Hz, duration: 12 h). We subjected the control cells to identical conditions without CTS exposure (Supporting Informations and Methods S1).

### 2.4 Microarray Analysis

We analyzed the microarray dataset from the Gene Expression Omnibus (GEO) (accession number GSE70362), including expression profiles from eight normal AF tissue samples. Data were processed using the Robust multi‐array average method and analyzed for mechanoreceptor gene expression. We selected *PIEZO1*, *PIEZO2*, *TRPA1*, *TRPM8*, and *TRPV4* genes for further analysis (Supporting Informations and Methods S1).

### 2.5 RNA Interference

RNA interference (RNAi) experiments were performed using synthetic small interfering RNA (siRNA) reagents from Dharmacon (Lafayette, CO, USA). Reverse transfection was performed using Lipofectamine RNAiMAX (Thermo Fisher Scientific, Waltham, MA, USA, Cat. #STEM00015) following the manufacturer's instructions. We assessed knockdown efficiency 48 h post‐transfection via RT‐qPCR, confirming an efficiency of ≥ 80%. We used the following siRNA constructs: si*Negative Control* (si*NC*), si*Piezo1*, si*Piezo2*, and si*Trpv4* (Supporting Informations and Methods S1).

### 2.6 Calcium Assay

We measured intracellular calcium responses using the calcium indicator dye Fluo‐8 NW (AAT Bioquest, Pleasanton, CA, Cat. #36315) and PIEZO1 agonist Yoda1 (Selleck, Houston, TX, Cat. #S6678). FlexStation 3 (Molecular Devices, San Jose, CA) was used to record fluorescence intensity changes. We calculated the maximum fluorescence intensity change (Δ*F*/*F*_0_) relative to the control data (Supporting Informations and Methods S1).

### 2.7 Real‐Time Quantitative PCR

RNA was extracted using the Direct‐zol RNA Microprep Kit (Zymo Research, Irvine, CA, Cat. #R2062). We performed reverse transcription of RNA to complementary DNA using the PrimeScript RT Master Mix (Takara Bio, Shiga, Japan, Cat. #RR036A). Real‐time quantitative PCR (RT‐qPCR) was performed using the QuantStudio 1 Real‐Time PCR System (Thermo Fisher Scientific) and Brilliant III Ultra‐Fast SYBR Green QPCR Master Mix (Agilent Technologies, Santa Clara, CA, Cat. #600882). Target gene primers were designed using NCBI Primer‐BLAST, and primer sequences are listed in Supporting Information Tables S2 and S3. Subsequently, relative gene expression levels were normalized to the gene glyceraldehyde‐3‐phosphate dehydrogenase (Gapdh) and calculated using the ΔΔCt method. Detailed methods and primer sequences are provided in the Supporting Informations and Methods S1.

### 2.8 RNA Sequencing

For CTS experiments, AF cells were subjected to moderate CTS (2% strain, 1 Hz) for 12 h, while non‐stretched cells served as controls. For Yoda1 treatment, AF cells were treated with 10 μM Yoda1 or DMSO for 12 h for the Yoda1 treatment. The total RNA was extracted following the same protocol described above. We assessed RNA integrity using TapeStation4150 (Agilent Technologies), and samples with an RNA integrity number (RIN) ≥ 8.0 were used for RNA sequencing (RNA‐seq) performed by AZENTA Life Sciences (Burlington, MA) using four biological replicates per group for the CTS experiment (control and CTS groups, *n* = 4 each) and three biological replicates per group (DMSO and Yoda1 groups, *n* = 3 each). Differentially expressed gene (DEG) analysis (adjusted *p* < 0.1), Gene ontology (GO) analysis, and gene set enrichment analysis (GSEA) were performed using RStudio (v2025.09.2 + 418) (Supporting Informations and Methods S1).

### 2.9 Western Blotting

Nuclear proteins were extracted using the EPIXTRACT Nuclear Protein Isolation Kit II (Enzo Life Sciences, Farmingdale, NY, Cat. #ENZ‐45015). We quantified the protein concentration using the Bradford Protein Assay Kit (Takara Bio, Cat. #T9310A). The following antibodies were used: Runx2 (1:1000 dilution, Rabbit Anti‐Runx2, Cell Signaling Technology, Danvers, MA, Cat. #12556), F‐Actin (Anti‐Actin hFAB Rhodamine, Bio‐Rad, Cat. #12004163), and IRDye goat anti‐rabbit IgG (LI‐COR Biosciences, Lincoln, NE, Cat. #926–68 071). The Odyssey Fc Imaging System (LI‐COR Biosciences) was used to detect immunoreactive proteins. Protein expression levels were normalized to F‐Actin (Supporting Informations and Methods S1).

### 2.10 Osteogenic Differentiation

We induced osteogenic differentiation by treating cells with recombinant human bone morphogenetic protein 2 (BMP2, R&D Systems, Minneapolis, MN, Cat. #355‐BEC‐010) every 72 h. The control group received an equivalent volume of 4 mM HCl instead of BMP2. We assessed osteogenic differentiation using RT‐qPCR after 2 weeks, Alizarin Red staining after 3 weeks, and immunocytochemistry for phospho‐Smad1/5/9 (p‐Smad1/5/9, Cell Signaling, Cat. #13820). The concentrations of administered reagents were as follows: BMP2 (100 ng/mL), Yoda1 (10 μM), cyclosporin A (CsA; 100 nM, Fujifilm, Tokyo, Japan; Cat. #031–24 931), and ionomycin (1 μM, Cayman Chemical, Ann Arbor, MI; Cat. #10004974). Detailed procedures are provided in the Supporting Informations and Methods S1.

### 2.11 Calcineurin Phosphatase Activity Assay

Calcineurin enzymatic activity was measured using a calcineurin cellular activity assay kit (Enzo Life Sciences; Cat. #BML‐AK816) according to the manufacturer's instructions. AF cells were treated with Yoda1 (10 μM) with or without CsA (100 nM, 30 min pretreatment) for 30 min, lysed using the supplied extraction buffer, and clarified by centrifugation. After removal of free phosphate, equal amounts of protein were incubated with the RII phosphopeptide substrate at 30°C for 30 min. Reactions were terminated by addition of BIOMOL Green reagent. Absorbance was measured at 620 nm, and calcineurin activity was calculated using a phosphate standard curve.

### 2.12 Statistical Analyses

All experiments were performed with biological replicates (*n* = 3 unless otherwise indicated), and each biological sample was measured in technical duplicate. Technical replicates were averaged and treated as a single data point; statistical analyses were performed using biological replicates only. Because normality testing is underpowered with *n* = 3, no normality tests were used to justify the statistical approach. For comparisons in which control and treated conditions were obtained from the same biological replicate, paired *t*‐tests were used. Importantly, the direction of change was consistent across all biological replicates (3/3), supporting the reproducibility of the observed trend. Ordinary one‐way analysis of variance (ANOVA) followed by Tukey's multiple comparisons test was used to compare three or more groups. Statistical significance was set at *p* < 0.05, with significance levels denoted as follows: *p < 0.05, **p < 0.01, ***p < 0.001, ****p < 0.0001. All analyses were performed using GraphPad Prism 10 (v.10.3.1, GraphPad Software, San Diego, CA, USA).

## 3 Results

### 3.1 Suppression of Ossification via PIEZO1‐Mediated Low‐Strain CTS in Rat AF Cells

**FIGURE 1:**
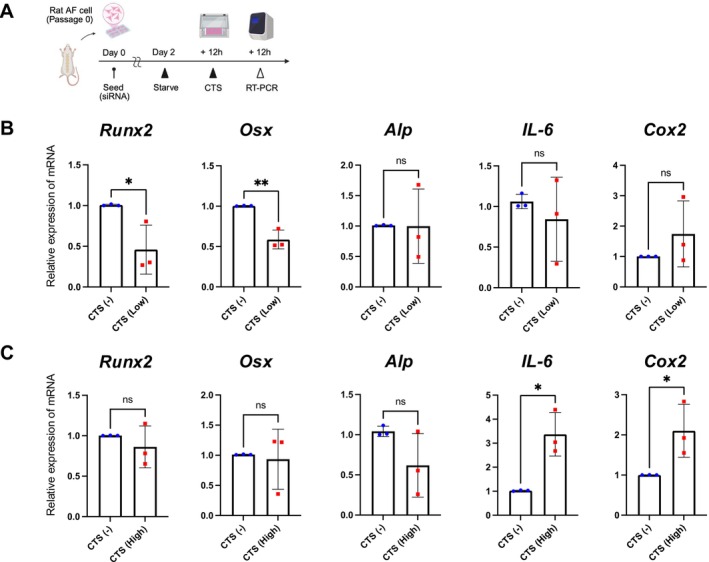
Suppression of osteogenic gene expression by low‐strain CTS in AF cells. (A) Schematic illustration of the experimental schedule for applying CTS to AF cells for 12 h. (B) RT‐qPCR analysis showing significant *Runx2* and *Osx* downregulation under low‐strain CTS. (C) In contrast, high‐strain CTS did not induce osteogenic gene downregulation but significantly increased *Il‐6* and *Cox2*. Data are presented as mean ± standard deviation (SD). Each group was compared with the control group (N‐CTS: Without CTS group). Statistical significance was set at *p* < 0.05, with significance levels denoted as follows: *p < 0.05, **p < 0.01, ***p < 0.001, ****p < 0.0001.

**FIGURE 2:**
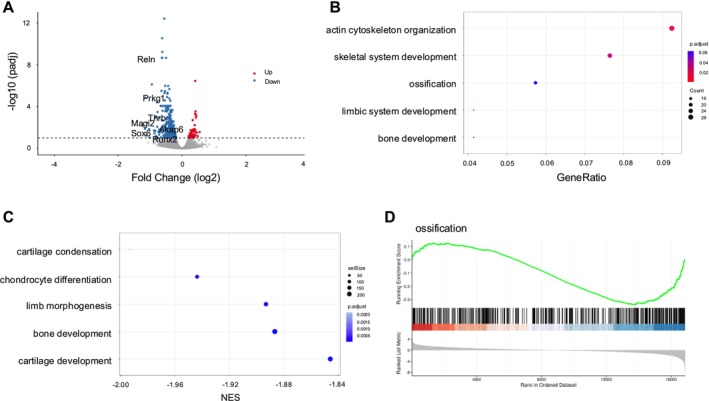
Transcriptomic analysis of AF cells subjected to moderate CTS. (A) Volcano plot of DEGs in AF cells exposed to moderate CTS, showing 39 upregulated and 326 downregulated genes. (B) GO enrichment analysis of downregulated genes identified multiple biological processes, including terms related to ossification and skeletal system development. Bone‐ and cartilage‐related GO terms were extracted from the full enrichment list and visualized. (C) GSEA using the ranked full gene list revealed negative enrichment of ossification‐related gene sets under moderate CTS. (D) Representative GSEA enrichment plot for the GO term “ossification,” showing negative enrichment in AF cells subjected to moderate CTS.

To investigate the effects of mechanical stimulation on AF cells, we compared gene expression changes between low‐ (2%) and high‐strain (12%) CTS for 12 h, and gene expression levels were evaluated using RT‐qPCR (Figure 1A). Under low‐strain stimulation, the expression levels of *Runx2* and *Osterix* (*Osx*), key transcription factors in osteogenesis, were significantly downregulated (Figure 1B). In contrast, high‐strain stimulation did not induce this downregulation but significantly increased pro‐inflammatory cytokine levels (*Il‐6* and *Cox2*) (Figure 1C). These findings indicate that low‐strain stimulation exerts an inhibitory effect on ossification in AF cells. To further clarify the global transcriptional effects of moderate mechanical stimulation, we performed RNA‐seq analysis of rat AF cells subjected to 2% CTS. Moderate CTS induced substantial transcriptional changes, with 326 genes downregulated and 39 genes upregulated (Figure 2A). GO enrichment analysis identified multiple downregulated biological processes, including terms related to ossification and skeletal development, consistent with the biological focus of our study (Table S4). Based on these results, bone‐ and cartilage‐related GO terms were extracted from the full enrichment dataset and visualized (Figure 2B). GSEA using the ranked full gene list revealed negative enrichment of ossification‐related gene sets under moderate CTS, in agreement with the suppression of osteogenic programs observed in the GO analysis (Table S5; Figure 2C,D).

To identify potential mechanosensitive channels involved in this response in AF cells, we first examined mechanoreceptor gene expression in rat AF cells using RNA‐seq data from the CTS‐minus (control) group. This analysis revealed that multiple mechanoreceptor genes were expressed in rat AF cells, among which *Piezo1* and transient receptor potential vanilloid 4 (*Trpv4*)—mechanoreceptors previously reported to play functional roles in musculoskeletal tissues—exhibited relatively higher basal expression compared with other mechanoreceptors (Figure S1A). To determine whether a similar expression pattern is conserved in human tissue, we next analyzed mechanoreceptor gene expression in normal human AF tissue using publicly available microarray data (GEO Accession number: GSE70362) [38]. Consistent with the rat data, multiple mechanoreceptor genes were expressed in normal human AF tissue, with PIEZO1 and TRPV4 showing relatively higher expression levels compared with other mechanoreceptors (Supplementary Figure S1B).

**FIGURE 3:**
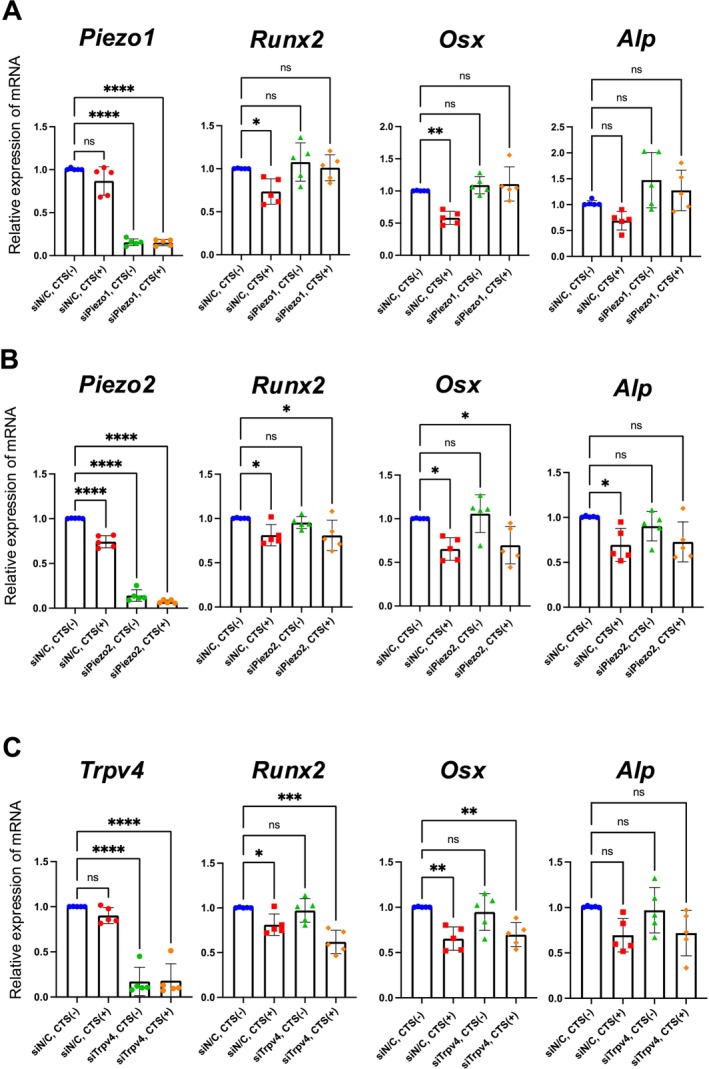
Piezo1 mediates the suppression of osteogenic gene expression under low‐strain CTS. The knockdown efficiency was confirmed to be approximately 80% for each gene. (A) RT‐qPCR showed that si*Piezo1* reversed the low‐strain CTS‐induced downregulation of *Runx2* and *Osx*. (B, C) si*Piezo2* or si*Trpv4* did not reverse osteogenic gene suppression by low‐strain CTS. An ordinary one‐way analysis of variance (ANOVA), followed by Tukey's multiple comparisons test was used. Each group was compared with the siN/*C* without the CTS group. Statistical significance was set at *P* < 0.05, with significance levels denoted as follows: *P < 0.05, **P < 0.01, ***P < 0.001, ****P < 0.0001.

Next, we evaluated the functional involvement of candidate mechanoreceptors in low‐strain–induced suppression of ossification based on their basal expression levels and reported mechanobiological relevance. RNAi‐mediated knockdown of *Piezo1*, *Piezo2*, a member of the PIEZO family, and *Trpv4*, followed by low‐strain CTS. The knockdown efficiency for all genes was approximately 80% (Figure 3). In the control (si*NC*) group, low‐strain CTS significantly reduced *Runx2* and *Osx* expression, but this downregulation was suppressed in the si*Piezo1* group (Figure 3A). In contrast, the knockdown of *Piezo2* (si*Piezo2*) and *Trpv4* (si*Trpv4*) did not prevent CTS‐induced downregulation of *Runx2* and *Osx* (Figure 3B,C), indicating that the inhibitory effect of low‐strain stimulation on ossification in AF cells is predominantly mediated through Piezo1.

### 3.2 Inhibition of Osteogenic Gene Expression via PIEZO1 Activation in Rat and Human AF Cells

**FIGURE 4:**
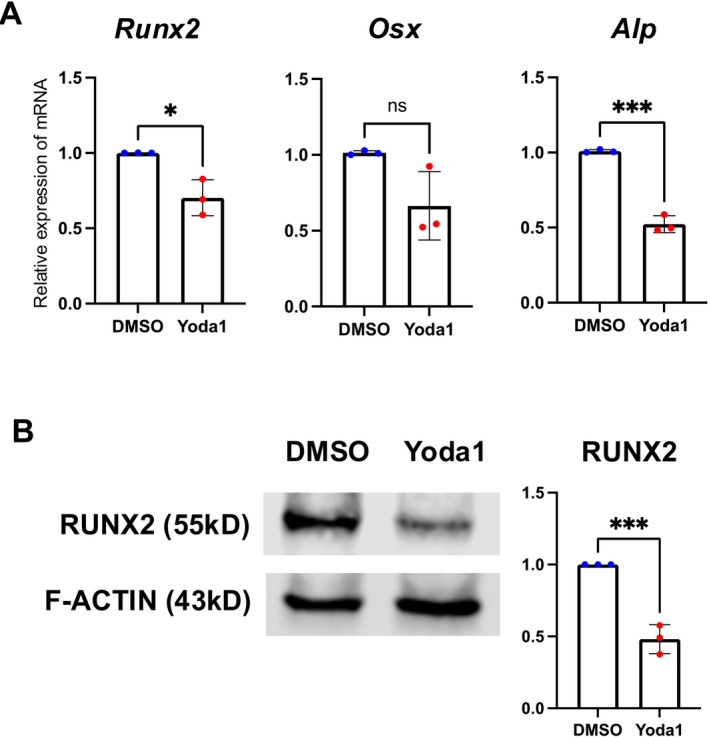
Activation of Piezo1 by Yoda1 suppresses osteogenic markers in rat AF cells. (A) RT‐qPCR showed downregulation of *Runx2*, *Osx*, and *Alp* after treatment with 10 μM Yoda1 for 12 h. (B) Western blotting analysis revealed that RUNX2 protein levels decreased in rat AF cells after 24‐h Yoda1 treatment. Figure S3 shows the full‐length blot images. Statistical significance was set at *P* < 0.05, with significance levels denoted as follows: *P < 0.05, **P < 0.01, ***P < 0.001, ****P < 0.0001.

**FIGURE 5:**
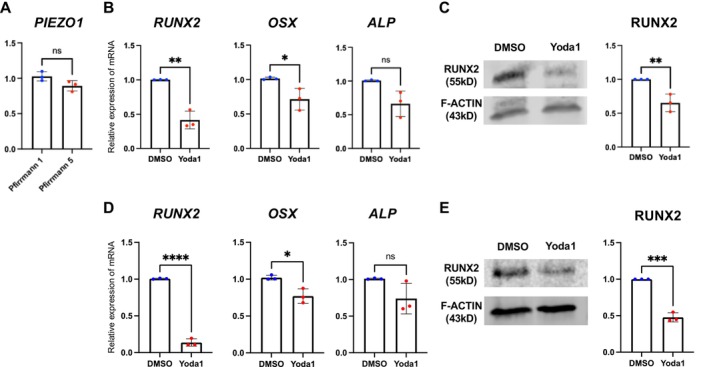
Activation of Piezo1 by Yoda1 suppresses osteogenic markers in human AF cells. Human AF cells were isolated from nondegenerated (Pfirrmann grade 1) and severely degenerated (Pfirrmann grade 5) intervertebral discs. (A) PIEZO1 mRNA expression showed no significant difference between grade 1 and grade 5 cells. (B–E) Consistent with observations in rat AF cells, treatment with Yoda1 (10 μM, 12 h) significantly downregulated osteogenesis‐related genes (*RUNX2*, *OSX*) at the mRNA level and reduced RUNX2 protein expression. Full‐length Western blot images are shown in Figure S3. Statistical significance was set at *P* < 0.05, with significance levels denoted as follows: *P < 0.05, **P < 0.01, ***P < 0.001, ****P < 0.0001.

To mimic PIEZO1 activation induced by mechanical stimulation, Yoda1, a PIEZO1 agonist, was administered to AF cells at 1, 5, 10, 25, and 50 μM, and intracellular calcium influx was measured, resulting in a significant increase in the intracellular calcium influx at ≥ 10 μM (Figure S2). Based on this result, RNA was extracted from AF cells treated with 10 μM Yoda1 for 12 h, followed by RT‐qPCR analysis, which downregulated osteogenic genes (*Runx2*, *Osx*, and *Alp*), similar to the effects observed under low‐strain CTS (Figure 4A). RUNX2 protein levels were examined by Western blotting to further validate these findings, confirming a significant reduction following Yoda1 treatment (Figure 4B). We then conducted comparable experiments using human AF cells isolated from intervertebral discs of patients with nondegenerated (Pfirrmann grade 1) and severely degenerated (Pfirrmann grade 5) discs. No significant difference in PIEZO1 mRNA expression was observed between grade 1 and grade 5 tissues (Figure 5A). Consistent with the results in rat AF cells, Yoda1 treatment significantly downregulated osteogenesis‐related genes (*RUNX2*, *OSX*) at the mRNA level and reduced RUNX2 protein expression (Figure 5B–E). Together, these results demonstrate that the inhibitory effect of PIEZO1 activation on osteogenic signaling is observed in rat and human AF cells. Figure S3 shows the original Western blotting images.

**FIGURE 6:**
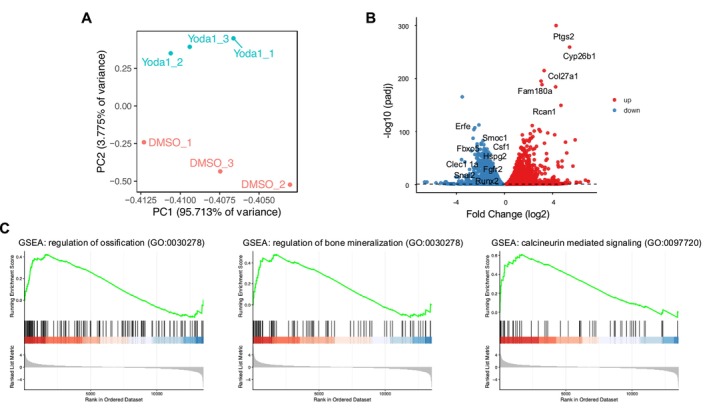
Transcriptomic analysis of AF cells following Piezo1 activation by Yoda1. (A) PCA showed a clear separation between the control (DMSO) and Yoda1‐treated groups. (B) Volcano plot of DEGs shows 3281 upregulated and 3362 downregulated genes. (C) GSEA reveals enrichment of ossification‐ and calcium signaling‐related pathways, including regulation of ossification, regulation of bone mineralization, and calcineurin‐mediated signaling, in Yoda1‐treated AF cells.

RNA‐seq was performed to comprehensively evaluate the mechanism underlying the ossification‐inhibitory effect of Piezo1 activation in AF cells. Three biological replicates were analyzed for the control (DMSO) and Yoda1‐treated groups. Principal component analysis demonstrated distinct clustering between the two groups, indicating markedly different transcriptomic profiles (Figure 6A). DEG analysis identified 3281 upregulated and 3362 downregulated genes between the groups (Figure 6B). GO analysis showed that genes downregulated by PIEZO1 activation were predominantly associated with cell cycle‐ and chromosome‐related processes, whereas upregulated genes were enriched in autophagy‐ and vesicle‐related pathways. These results suggest that PIEZO1 activation induces a transcriptional shift from proliferative programs toward intracellular regulatory and signaling processes in AF cells (Figure S4; Table S6). Furthermore, GSEA identified multiple ossification‐related terms in the Yoda1‐treated group. Among enriched signaling pathways, calcineurin‐mediated signaling, which is activated by calcium influx, was notably enriched (Figure 6C; Table S7), indicating that intracellular calcium influx through Piezo1 activation influences the signaling pathways involved in ossification, further supporting the role of Piezo1 in regulating ossification processes in AF cells. To assess whether ossification‐related transcriptional changes induced by PIEZO1 activation are shared with mechanical stimulation, we compared RNA‐seq datasets from CTS‐treated and Yoda1‐treated AF cells. This analysis identified 135 commonly downregulated genes, nine of which—including *Runx2*—were annotated to ossification‐related GO terms. GSEA of this shared gene set demonstrated significant enrichment of the ossification regulation pathway (Figure S5). These findings indicate that both mechanical stimulation and pharmacological activation of PIEZO1 induce overlapping transcriptional programs associated with ossification suppression in AF cells.

### 3.3 Regulation of the BMP2/SMAD Pathway via Calcineurin Signaling Activation by PIEZO1

**FIGURE 7:**
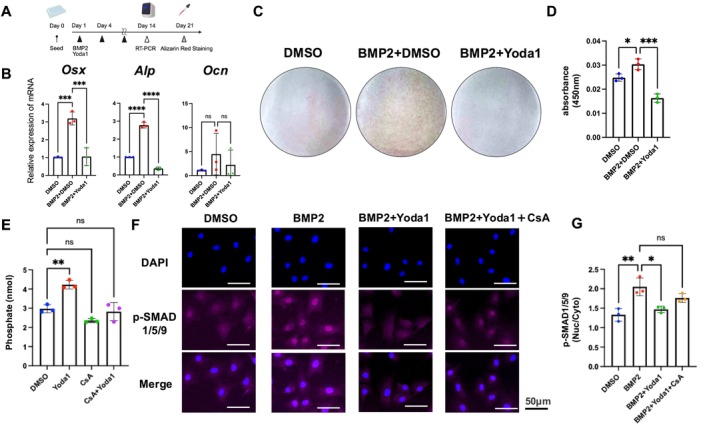
Piezo1 activation inhibits BMP2‐induced osteogenesis through calcineurin signaling. (A) Schematic of the experimental schedule for the BMP2 and Yoda1 treatment. (B) RT‐qPCR showed that Yoda1 cotreatment suppressed the BMP2‐induced upregulation of Osx and Alp in AF cells. (C, D) Alizarin Red staining revealed that BMP2 alone enhanced calcium deposition, whereas cotreatment with Yoda1 reduced this staining intensity. (E) Calcineurin phosphatase assay showing that Yoda1 significantly increased calcineurin enzymatic activity compared with DMSO control; this effect was abolished by co‐treatment with cyclosporin A (CsA). (F, G) Immunocytochemistry for p‐Smad1/5/9 showed increased nuclear translocation after BMP2 treatment but significantly decreased BMP2 + Yoda1 cotreated group. Furthermore, cotreatment with the calcineurin inhibitor CsA rescues the nuclear translocation of p‐Smad1/5/9. Statistical significance was set at *P* < 0.05, with significance levels denoted as follows: *P < 0.05, **P < 0.01, ***P < 0.001, ****P < 0.0001.

We examined whether Yoda1 could suppress BMP2‐induced osteogenesis to determine whether PIEZO1 activation exerts an anti‐ossification effect in AF cells (Figure 7A). RT‐qPCR analysis showed that BMP2 treatment significantly upregulated the expression of osteogenic markers *Osx* and *Alp*. However, cotreatment with Yoda1 markedly suppressed the expression of these genes (Figure 7B). Furthermore, Alizarin Red staining performed after 3 weeks of continuous treatment revealed that BMP2 alone enhanced calcium deposition, whereas cotreatment with Yoda1 attenuated this mineralization (Figure 7C,D), indicating that PIEZO1 activation by Yoda1 inhibits BMP2‐induced osteogenesis at the transcriptional and protein levels, supporting its regulatory role in bone formation within AF cells.

**FIGURE 8:**
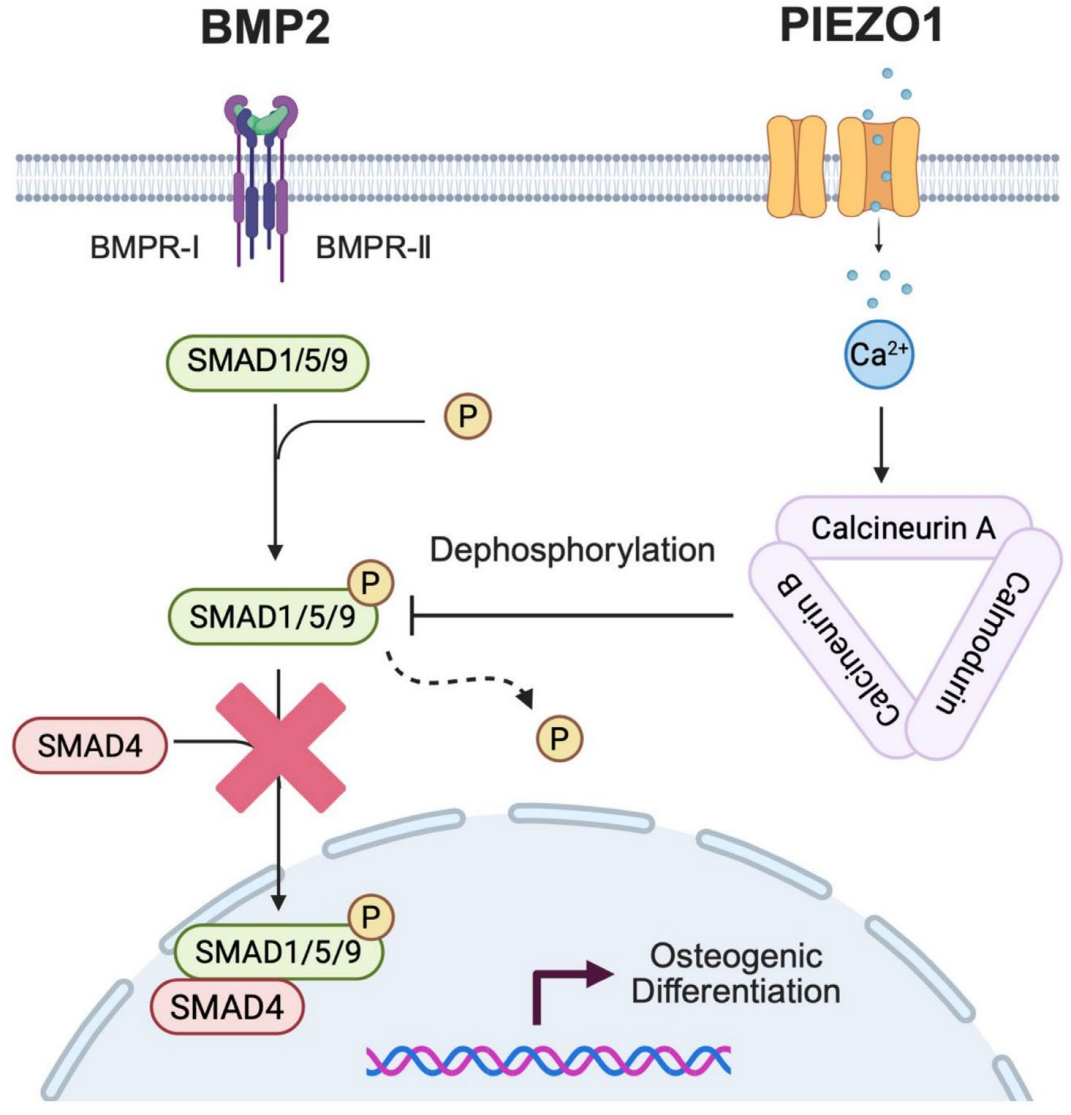
Proposed mechanism schema. Piezo1‐mediated calcium influx activates calcineurin, which dephosphorylates p‐Smad1/5/9, inhibiting its nuclear translocation and BMP2 signaling.

Our previous RNA‐seq analysis results indicated the involvement of calcineurin signaling in the downstream response to PIEZO1 activation (Figure 6D). Calcineurin is a calcium‐dependent serine/threonine protein phosphatase that negatively regulates BMP2/SMAD signaling by directly dephosphorylating p‐SMAD1/5/9 through its interaction with calcineurin A and B with calmodulin [39, 40]. A calcineurin phosphatase assay was used to directly evaluate whether PIEZO1 activation enhances calcineurin activity in AF cells. Yoda1 treatment significantly increased calcineurin enzymatic activity compared with the DMSO control, indicating that PIEZO1 stimulation activates the calcineurin pathway. This increase was abolished by co‐treatment with CsA, confirming that the observed phosphatase activity is calcineurin‐dependent (Figure 7E). To further assess the effects of Piezo1 activation on this pathway, immunocytochemistry was performed to examine the nuclear translocation of p‐Smad1/5/9 in response to BMP2. The nuclear translocation of p‐Smad1/5/9 was significantly increased after 1 h of BMP2 treatment, but it was substantially reduced in the BMP2 plus Yoda1 group. Furthermore, co‐treatment with cyclosporin A (CsA), a calcineurin inhibitor, reversed the suppressive effect of Yoda1 on p‐SMAD1/5/9 nuclear translocation (Figure 7F,G). Ionomycin, a pharmacological calcineurin activator, was used to determine whether calcineurin activation alone is sufficient to reproduce the inhibitory effect observed with Yoda1. Under BMP2 co‐treatment, ionomycin similarly reduced nuclear translocation of p‐SMAD1/5/9 (Figure S6). These results closely parallel those observed with Yoda1, supporting the conclusion that calcineurin activation mediates suppression of BMP2/SMAD1/5/9 signaling. Collectively, these findings indicate that Piezo1 activation promotes intracellular calcium influx, activating calcineurin signaling. This, in turn, dephosphorylates p‐Smad1/5/9 and prevents its nuclear translocation, thereby inhibiting BMP2 signaling (Figure 8).

## 4 Discussion

We demonstrated that low‐intensity stretch stimulation mediated by Piezo1 suppresses osteogenic‐related gene expression in rat AF cells. Specifically, PIEZO1 activation engages the calcineurin signaling pathway, which inhibits the BMP/SMAD pathway, ultimately suppressing ossification.

Although PIEZO1 is expressed throughout the AF, NP, and cartilaginous endplate (CEP) (Figure S7), its functional roles differ among these tissues. Recent studies have reported that PIEZO1 activation in the CEP promotes osteogenic and calcific changes that accelerate disc degeneration. Peng et al. demonstrated that PIEZO1 overexpression aggravates CEP ossification via the Ca^2+^/F‐actin/YAP axis [41]. Similarly, Lin et al. showed that PIEZO1 enhances CEP chondrocyte senescence and apoptosis through the Ca^2+^/CaMKII/Drp1 pathway, accompanied by increased RUNX2 expression, leading to endplate calcification [42]. In contrast, PIEZO1 in the NP and AF primarily mediates oxidative stress, mitochondrial dysfunction, and matrix catabolism rather than directly inducing osteogenesis [30, 43]. Thus, PIEZO1‐dependent osteogenic or calcific effects appear tissue‐specific, being most pronounced in the CEP. However, its role in regulating ossification within the AF remains unclear. To address this gap, our study delineated AF‐specific consequences of PIEZO1 activation and clarified its role in osteogenic regulation.

As a structural component of IVD, the AF is exposed to a complex in vivo mechanical environment that includes compression, shear, bending, and tension—all of which dynamically fluctuate during spinal motion and loading. Among these forces, AF tissue encounters substantial circumferential tensile forces that arise from the hydrostatic pressure generated by the NP. These tensile forces are especially pronounced in the outer and posterior AF regions [44]. Previous studies have demonstrated that the AF experiences cyclic tensile loading in vivo and that circumferential tension is the predominant mechanical mode of the AF [45]. For these reasons, CTS is an appropriate and widely used in vitro model for investigating AF cell mechanobiology. While CTS does not fully replicate the multifactorial loading environment—including compressive and shear forces—it provides a controlled, reproducible, and physiologically relevant mechanical stimulus that captures a principal loading mode experienced by AF tissue. The biological effect of tensile strain depends on its magnitude, frequency, and duration [46]. Our study showed that low‐intensity CTS significantly downregulated osteogenic markers in AF cells, whereas high‐intensity CTS failed to elicit this effect and instead induced inflammatory responses. This is consistent with prior work indicating that mechanical overload preferentially triggers inflammatory responses in AF cells. Notably, high tensile strain has been reported to activate MAPK signaling, suppress proliferation and migration, and upregulate inflammatory genes in human AF cells [22, 25]. Chen et al. further demonstrated that applying high cyclic stretching (15% strain, 1 Hz) in human AF cells upregulated the expression of osteogenic markers, such as RUNX2, OSX, osteopontin, and BMP2, whereas low cyclic stretching (5% strain, 1 Hz) did not induce these changes. Furthermore, high‐intensity mechanical stimulation promotes BMP2 expression and enhances osteogenesis through the p38 MAPK and BMP/SMAD signaling pathways [13]. In contrast, 12% CTS in our system did not significantly induce osteogenic markers, likely reflecting differences in experimental context, including species, degeneration status, and passage number, as prior studies typically used human AF cells from severely degenerated discs expanded through multiple passages [13]. Together, these findings support a threshold‐dependent or biphasic mechanobiological response of AF cells that is characterized by adaptive signaling under physiological strain and pathological responses under mechanical overload.

Generally, low‐intensity stimulation enhances anabolic responses in AF cells [24, 25, 47]. Consistent with this concept, our study showed that 2% strain at 1 Hz—approximating physiological frequency during walking [48]—reduced osteogenic‐related gene expression, indicating that moderate exercise may prevent ossification within the IVD, whereas disuse or excessive strain could disrupt this balance and promote pathological ossification or calcification. Although direct Ca^2+^ imaging was not performed during CTS, PIEZO1‐mediated Ca^2+^ influx is known to be stimulus‐intensity dependent [36]. Under low‐to‐moderate strain, we speculate that PIEZO1 activation induces transient Ca^2+^ signaling that preferentially engages adaptive pathways, such as calcineurin‐dependent suppression of BMP2/SMAD signaling, to maintain AF homeostasis. In contrast, higher strain may lead to sustained Ca^2+^ influx, channel adaptation, and a shift toward inflammatory or injurious responses.

Functional experiments showed that the knockdown of *Piezo1* (si*Piezo1*) abolished the osteogenesis‐suppressing effect of low‐intensity CTS, whereas the knockdown of *Piezo2* or *Trpv4* did not, indicating that Piezo1 plays a key role in transducing moderate mechanical signals to suppress osteogenic gene expression in AF cells. Although TRPV4 regulates water content and extracellular matrix remodeling in IVD tissues [49], and its deletion in mice accelerates disc degeneration, it does not participate in the anti‐ossification response under low mechanical strain [50]. Similarly, although PIEZO2 is essential for mechano‐sensation in neural tissues, it was not functionally relevant in this context [51].

In contrast, PIEZO1 is widely expressed in nonsensory tissues exposed to mechanical stress and plays roles in multiple organ systems, including the respiratory, circulatory, digestive, and urinary systems [28]. Within the musculoskeletal system, PIEZO1 has been implicated in bone [52], cartilage [53], muscle [54], and tendon biology [34]. PIEZO1 has been reported to promote osteogenic differentiation in various cell types, including mesenchymal stem cells and osteoblast‐lineage cells [52, 55]. However, our findings demonstrate that PIEZO1 activation in AF cells does not promote osteogenesis; instead, it suppresses it. This discrepancy likely reflects fundamental differences in cellular lineage and tissue‐specific mechanobiology. AF cells, as specialized fibrocartilaginous cells contiguous with spinal ligaments, must maintain a non‐mineralized extracellular matrix to preserve disc flexibility and prevent pathological calcification. These results indicate that PIEZO1 signaling in AF cells operates under a distinct mechanobiological set‐point: rather than acting as an osteogenic switch, moderate PIEZO1 activation serves as a protective brake that restrains BMP/SMAD‐driven osteogenic drift, maintaining AF homeostasis under physiological mechanical loading. From a translational perspective, modulating PIEZO1 activity may represent a novel strategy to prevent pathological ossification within intervertebral discs. Although PIEZO1 activation has been exploited to enhance bone formation in skeletal tissues, our data suggest that controlled activation in AF cells could suppress aberrant osteogenic signaling. Given the tissue‐ and intensity‐specific nature of PIEZO1 responses, and reports of PIEZO1‐mediated catabolic effects in NP cells, future studies are needed to define finely tuned modulation strategies that preserve disc homeostasis without adverse effects on adjacent musculoskeletal tissues.

BMP2 is a well‐known inducer of osteogenesis in AF cells exposed to high intensity strain [13]. Consistent with this, BMP2 enhanced osteogenic gene expression and matrix mineralization in our system. However, cotreatment with Yoda1 suppressed transcriptional and protein‐level indicators of osteogenesis. Because AF cells possess progenitor‐like properties and are capable of chondrogenic and osteogenic differentiation [13, 14], our findings indicate that PIEZO1 can shift the differentiation potential of these cells by inhibiting the BMP pathway. The BMP/SMAD signaling cascade is a major osteogenic pathway downstream of TGF‐β signaling. BMPs bind to type I and II serine/threonine kinase receptors, phosphorylating SMAD1/5/9, forming a complex with SMAD4 that translocates to the nucleus to regulate key osteogenic genes, including *RUNX2*, *OSX*, and *DLX5*, thereby promoting the differentiation of stem cells into osteoblasts and enhancing bone formation [56]. Nakamura et al. reported that Bmp2 and its receptors are co‐expressed in mouse AF and endplate tissues [15]. Interestingly, Yoda1 treatment tended to increase BMP2 mRNA expression in both rat and human AF cells (Figure S8) despite also suppressing downstream osteogenic markers. This dissociation suggests that PIEZO1 activation attenuates osteogenic signaling at a step downstream of BMP2 transcription, consistent with our observations for SMAD1/5/9 signaling. In our study, PIEZO1 activation attenuated BMP2‐induced nuclear translocation of p‐SMAD1/5/9. Calcineurin is known to negatively regulate BMP signaling by directly dephosphorylating p‐SMAD1/5/9 [39]. Using a calcineurin cellular activity assay, we confirmed that Yoda1 significantly increases calcineurin enzymatic activity, and this activation is inhibited by CsA. Imaging experiments further support this mechanism: ionomycin, a calcium‐dependent calcineurin activator, reproduced the inhibitory effect on BMP2‐induced p‐SMAD1/5/9 nuclear translocation, whereas CsA restored nuclear localization. Collectively, these data support a model in which PIEZO1‐mediated Ca^2+^ influx activates calcineurin, thereby attenuating BMP2‐induced nuclear translocation of p‐SMAD1/5/9 and ultimately suppressing osteogenic signaling in AF cells. Although further studies, including co‐immunoprecipitation, are needed to fully delineate this regulatory axis, our results identify calcineurin as a key downstream mediator of PIEZO1 signaling in the AF and a potential therapeutic target for preventing pathological disc ossification.

Although research on PIEZO1 in AF remains limited, PIEZO1 expression increased during degeneration in studies using a rat model. Additionally, when rat AF cells were exposed to cyclic mechanical stretch, apoptosis increases with strain magnitude (5%–20%), with a significant increase in Piezo1 expression at higher strain [43]. Differences in experimental conditions likely explain why enhanced PIEZO1 activation under high strain reported in earlier studies was not directly observed in our system (Figure S9). Specifically, prior work employed rat AF cells expanded to later passages and subjected them to prolonged high‐magnitude cyclic stretch, whereas we used primary (passage 0) rat AF cells and applied a high‐strain condition optimized to preserve cell adhesion. These distinctions suggest that PIEZO1‐dependent mechanotransduction in AF cells is influenced not only by strain magnitude but also by frequency, duration, cumulative loading cycles, cell passage number, and the extracellular matrix context. Integrating these considerations with our findings, we propose a dual role for PIEZO1 in AF cells: excessive activation under high strain promotes cell death and degeneration, whereas moderate activation preserves homeostasis and suppresses ossification.

Ossification within the AF has been observed in rat models of disc degeneration, accompanied by hypertrophic chondrocytes and osteoblasts [14]. Controlling aberrant ossification could therefore represent a promising strategy for maintaining AF structure and function. Our findings indicate that the moderate activation of PIEZO1 may serve such a role by limiting BMP2 signaling and downstream osteogenic differentiation. Thus, the pharmacological or mechanical modulation of PIEZO1 could emerge as a novel therapeutic approach for preventing ectopic ossification and mitigating intervertebral disc degeneration.

This study has several limitations. First, the relatively small sample size (*n* = 3 per group) may limit generalizability. Although consistent trends were observed across experiments, validation in larger cohorts is needed to confirm the robustness and reproducibility of these findings. Second, regarding the human AF cells used, Pfirrmann grade 5 discs were obtained from three different patients (three discs), whereas grade 1 discs were obtained from only two AIS patients (three discs). Consequently, age‐ and degeneration‐related biological variability cannot be completely excluded, and it is possible that degeneration influences PIEZO1 responsiveness even when baseline expression appears similar. Third, direct intracellular Ca^2+^ imaging during CTS could not be performed due to technical constraints. Notably, previous studies using laser‐induced shockwave stimulation have demonstrated that PIEZO1‐mediated Ca^2+^ influx increases in a stimulus‐intensity–dependent manner [36]. Finally, no in vivo experiments were conducted. Future studies employing localized Piezo1 modulation, such as AF‐targeted Yoda1 delivery or genetic activation models, are needed to validate whether the in vitro suppression of osteogenic drift by Piezo1 occurs in animal models of IVDD. Further investigations examining interactions between PIEZO1 and other mechanosensitive channels could also advance our understanding of disc mechanobiology.

## 5 Conclusion

We demonstrated that moderate mechanical loading mediated by PIEZO1 suppresses ossification, contributing to AF homeostasis. Mechanistically, PIEZO1 activation enhances calcineurin signaling, inhibiting the BMP/SMAD pathway and downstream osteogenic gene expression, indicating new insights into the pathophysiology of IVDD and highlighting PIEZO1 as a potential therapeutic target for disc degeneration and associated LBP.

## Author Contributions

Conception and design: Hisakazu Shitozawa, and Ryo Nakamichi. Collection and assembly of data: Hisakazu Shitozawa, Ryo Nakamichi, Aki Yoshida, and Masataka Ueda. Statistical analysis: Hisakazu Shitozawa. Interpretation of data: Hisakazu Shitozawa and Ryo Nakamichi. Drafting of the article: Hisakazu Shitozawa and Ryo Nakamichi. Acquisition of funding: Hisakazu Shitozawa and Ryo Nakamichi. Critical revision of the article: Ryo Nakamichi, Aki Yoshida, Masataka Ueda, Taichi Saito, Koji Uotani, Yoshiaki Oda, Ryo Takatori, Kazutaka Yamashita, and Toshifumi Ozak. Final approval of the article: Hisakazu Shitozawa, Ryo Nakamichi, Aki Yoshida, Masataka Ueda, Taichi Saito, Koji Uotani, Yoshiaki Oda, Ryo Takatori, Kazutaka Yamashita, and Toshifumi Ozak.

## Funding

This work was supported by Japan Society for the Promotion of Science (grant number 24K12307), Nakatomi Foundation (grant number 7102200377), and Naito Foundation (grant number 7102300454).

## Conflicts of Interest

The authors declare no conflicts of interest.

## Supporting information


**Figure S1:** Basal expression of mechanoreceptor genes in the AF tissue. (A) Baseline expression levels of representative mechanoreceptor genes in rat AF cells, assessed by RNA‐seq using samples from the CTS‐minus (control) group. Multiple mechanoreceptor genes were detectably expressed in rat AF cells, among which *Piezo1* and *Trpv4*—mechanoreceptors previously reported to play functional roles in musculoskeletal tissues—exhibited relatively higher basal expression levels compared with other candidates. (B) Gene expression profiling of mechanoreceptor genes in human AF tissue using publicly available microarray data (GEO accession number: GSE70362). Consistent with the rat data, *PIEZO1* and *TRPV4* showed higher expression levels than other mechanoreceptor genes in normal human intervertebral discs, supporting their potential involvement in mechanotransduction in AF tissue.

**Figure S2:** Intracellular calcium influx in AF cells following Piezo1 activation by Yoda1. AF cells were treated with increasing concentrations of Yoda1 (1, 5, 10, 25, and 50 μM), and intracellular calcium influx was measured using a calcium‐sensitive fluorescent dye. Calcium influx significantly increased at ≥ 10 μM, indicating the dose‐dependent activation of Piezo1 channels.

**Figure S3:** Full‐length images of the Western blotting shown in Figures 4B and 5C,E. Original, uncropped Western blotting images corresponding to the cropped panels in Figure 4B (rat AF cells), 5C and E (human AF cells) are presented. These images validate the reduction of RUNX2 protein expression after treatment with the Piezo1 agonist Yoda1. (A, B): rat, (C, D): human, Pfirrmann grade 1, (E, F): human, Pfirrmann grade 5.

**Figure S4:** GO analysis of Yoda1‐treated AF cells. GO enrichment analysis revealed that genes downregulated by Piezo1 activation were predominantly associated with cell cycle‐ and chromosome‐related processes, including chromosome organization and DNA replication. In contrast, upregulated genes were enriched in autophagy‐ and vesicle‐related pathways, such as regulation of autophagy and vesicle organization. These findings suggest that Piezo1 activation induces a transcriptional shift from proliferative programs toward intracellular regulatory and signaling processes in AF cells.

**Figure S5:** Comparison of transcriptomic responses to moderate CTS and pharmacological Piezo1 activation in AF cells. (A) Venn diagram illustrating the overlap of genes downregulated by moderate CTS and Yoda1 treatment. A total of 135 genes were commonly downregulated under both conditions. Among these, nine genes—including *Runx2*—were annotated to ossification‐related GO terms. (B) GSEA performed using the ranked gene list demonstrated significant enrichment of the GO term “regulation of ossification” among genes commonly downregulated by CTS and Yoda1 treatment. Together, these data indicate that mechanical stimulation and pharmacological activation of Piezo1 elicit partially overlapping transcriptional programs associated with suppression of ossification‐related pathways in AF cells.

**Figure S6:** Immunocytochemistry following Ionomycin treatment. Ionomycin was used as a pharmacological calcineurin activator. Under BMP2 co‐treatment, ionomycin reduced nuclear translocation of p‐Smad1/5/9, indicating calcineurin‐dependent suppression of BMP‐Smad signaling.

**Figure S7:** Immunohistochemistry of rat IVD. (A) FAST staining of rat IVD. (B) Immunohistochemistry for PIEZO1. PIEZO1 was expressed throughout the AF, NP, and CEP, with no appreciable difference in expression levels among these regions.

**Figure S8:** Bmp2 mRNA expression following Yoda1 treatment. Yoda1 treatment tended to increase BMP2 mRNA expression in both rat and human AF cells.

**Figure S9:** Piezo1 mRNA expression following CTS. Piezo1 expression was not significantly altered under either low‐ and high‐intensity CTS.

**Table S1:** Human sample metadata.
**Table S2:** Primer list of rats.
**Table S3:**. Primer list of humans.

**Table S4:** Full list of GO analysis under moderate CTS.

**Table S5:** Full list of GSEA under moderate CTS.

**Table S6:** Full list of GO analysis under Yoda1 treatment.

**Table S7:** Full list of GSEA under Yoda1 treatment.

**Data S1:** jsp270168‐sup‐0015‐supinfo.docx.

## Data Availability

All data generated in this study will be made publicly available. The raw RNA sequencing data (FASTQ files) have been deposited in the DDBJ Sequence Read Archive (DRA) under accession numbers PRJDB40026 and PRJDB20451 and are freely accessible without restriction. No additional documents will be provided.

## References

[1] N. Maniadakis and A. Gray , “The Economic Burden of Back Pain in the UK,” Pain 84 (2000): 95–103. 10601677 10.1016/S0304-3959(99)00187-6

[2] J. P. Urban and S. Roberts , “Degeneration of the Intervertebral Disc,” Arthritis Research & Therapy 5 (2003): 120–130. 12723977 10.1186/ar629 PMC165040

[3] S. Roberts , H. Evans , J. Trivedi , et al., “Histology and Pathology of the Human Intervertebral Disc,” Journal of Bone and Joint Surgery (American Volume) 88 (2006): 10–14. 10.2106/JBJS.F.00019 16595436

[4] M. K. Chelberg , G. M. Banks , D. F. Geiger , et al., “Identification of Heterogeneous Cell Populations in Normal Human Intervertebral Disc,” Journal of Anatomy 186 (1995): 43–53. 7544335 PMC1167271

[5] O. M. Torre , V. Mroz , M. K. Bartelstein , et al., “Annulus Fibrosus Cell Phenotypes in Homeostasis and Injury: Implications for Regenerative Strategies,” Annals of the New York Academy of Sciences 1442 (2019): 61–78. 30604562 10.1111/nyas.13964 PMC6417974

[6] P. Smits and V. Lefebvre , “Sox5 and Sox6 Are Required for Notochord Extracellular Matrix Sheath Formation, Notochord Cell Survival and Development of the Nucleus Pulposus of Intervertebral Discs,” Development 130 (2003): 1135–1148. 12571105 10.1242/dev.00331

[7] G. G. van den Akker , D. A. Surtel , A. Cremers , et al., “Novel Immortal Cell Lines Support Cellular Heterogeneity in the Human Annulus Fibrosus,” PLoS One 11 (2016): e0144497. 26794306 10.1371/journal.pone.0144497 PMC4721917

[8] N. Boos , S. Weissbach , H. Rohrbach , et al., “Classification of Age‐Related Changes in Lumbar Intervertebral Discs: 2002 Volvo Award in Basic Science,” Spine (Philadelphia, PA) 27 (2002): 2631–2644. 10.1097/00007632-200212010-00002 12461389

[9] J. A. Buckwalter , “Aging and Degeneration of the Human Intervertebral Disc,” Spine 20 (1995): 1307–1314. 7660243 10.1097/00007632-199506000-00022

[10] A. Prescher , “Anatomy and Pathology of the Aging Spine,” European Journal of Radiology 27 (1998): 181–195. 9717634 10.1016/s0720-048x(97)00165-4

[11] K. Chanchairujira , C. B. Chung , J. Y. Kim , et al., “Intervertebral Disk Calcification of the Spine in an Elderly Population: Radiographic Prevalence, Location, and Distribution and Correlation With Spinal Degeneration,” Radiology 230 (2004): 499–503. 14752191 10.1148/radiol.2302011842

[12] C. H. Yeh , L. Jin , F. Shen , G. Balian , and X. J. Li , “Mir‐221 Attenuates the Osteogenic Differentiation of Human Annulus Fibrosus Cells,” Spine Journal 16 (2016): 896–904. 10.1016/j.spinee.2016.03.026 PMC4970913 26997108

[13] C. N. Chen , H. I. Chang , C. K. Yen , W.‐L. Liu , and K.‐Y. Huang , “Mechanical Stretch Induced Osteogenesis on Human Annulus Fibrosus Cells Through Upregulation of Bmp‐2/6 Heterodimer and Activation of p38 and smad1/5/8 Signaling Pathways,” Cells 11 (2022): 2600. 36010676 10.3390/cells11162600 PMC9406707

[14] L. Jin , Q. Liu , P. Scott , et al., “Annulus Fibrosus Cell Characteristics Are a Potential Source of Intervertebral Disc Pathogenesis,” PLoS One 9 (2014): e96519. 24796761 10.1371/journal.pone.0096519 PMC4010482

[15] Y. Nakamura , H. Nakaya , N. Saito , and S. Wakitani , “Coordinate Expression of Bmp‐2, Bmp Receptors and Noggin in Normal Mouse Spine,” Journal of Clinical Neuroscience 13 (2006): 250–256. 16503488 10.1016/j.jocn.2005.05.011

[16] A. M. Hollenberg , N. Maqsoodi , A. Phan , et al., “Bone Morphogenic Protein‐2 Signaling in Human Disc Degeneration and Correlation to the Pfirrmann MRI Grading System,” Spine Journal 21 (2021): 1205–1216. 10.1016/j.spinee.2021.03.002 PMC8356724 33677096

[17] D. Haschtmann , S. J. Ferguson , and J. V. Stoyanov , “BMP‐2 and TGF‐beta3 Do Not Prevent Spontaneous Degeneration in Rabbit Disc Explants but Induce Ossification of the Annulus Fibrosus,” European Spine Journal 21 (2012): 1724–1733. 22639297 10.1007/s00586-012-2371-3 PMC3459107

[18] P. Cazzanelli and K. Wuertz‐Kozak , “Micrornas in Intervertebral Disc Degeneration, Apoptosis, Inflammation, and Mechanobiology,” International Journal of Molecular Sciences 21 (2020): 3601. 32443722 10.3390/ijms21103601 PMC7279351

[19] I. A. Stokes and J. C. Iatridis , “Mechanical Conditions That Accelerate Intervertebral Disc Degeneration: Overload Versus Immobilization,” Spine (Phila Pa 1976) 29 (2004): 2724–2732. 15564921 10.1097/01.brs.0000146049.52152.da PMC7173624

[20] J. Rainville , C. Hartigan , E. Martinez , et al., “Exercise as a Treatment for Chronic Low Back Pain,” Spine Journal 4 (2004): 106–115. 10.1016/s1529-9430(03)00174-8 14749199

[21] F. Rannou , T. S. Lee , R. H. Zhou , et al., “Intervertebral Disc Degeneration: The Role of the Mitochondrial Pathway in Annulus Fibrosus Cell Apoptosis Induced by Overload,” American Journal of Pathology 164 (2004): 915–924. 14982845 10.1016/S0002-9440(10)63179-3 PMC1613264

[22] H. Pratsinis , A. Papadopoulou , C. Neidlinger‐Wilke , et al., “Cyclic Tensile Stress of Human Annulus Fibrosus Cells Induces MAPK Activation: Involvement in Proinflammatory Gene Expression,” Osteoarthritis and Cartilage 24 (2016): 679–687. 26687822 10.1016/j.joca.2015.11.022

[23] H. T. Gilbert , J. A. Hoyland , A. J. Freemont , et al., “The Involvement of Interleukin‐1 and Interleukin‐4 in the Response of Human Annulus Fibrosus Cells to Cyclic Tensile Strain: An Altered Mechanotransduction Pathway With Degeneration,” Arthritis Research & Therapy 13 (2011): 1–12. 10.1186/ar3229 PMC3241352 21276216

[24] G. Sowa and S. Agarwal , “Cyclic Tensile Stress Exerts a Protective Effect on Intervertebral Disc Cells,” American Journal of Physical Medicine & Rehabilitation 87 (2008): 537–544. 18209665 10.1097/PHM.0b013e31816197ee PMC2935294

[25] W. Zhang , H. Wang , Z. Yuan , et al., “Moderate Mechanical Stimulation Rescues Degenerative Annulus Fibrosus by Suppressing Caveolin‐1 Mediated Pro‐Inflammatory Signaling Pathway,” International Journal of Biological Sciences 17 (2021): 1395–1412. 33867854 10.7150/ijbs.57774 PMC8040478

[26] R. Xiao and X. Z. Xu , “Mechanosensitive Channels: In Touch With Piezo,” Current Biology 20 (2010): R936–R938. 21056836 10.1016/j.cub.2010.09.053 PMC3018681

[27] P. Jin , L. Y. Jan , and Y. N. Jan , “Mechanosensitive Ion Channels: Structural Features Relevant to Mechanotransduction Mechanisms,” Annual Review of Neuroscience 43 (2020): 207–229. 10.1146/annurev-neuro-070918-050509 32084327

[28] D. Zhu , G. Zhang , X. Guo , et al., “A New Hope in Spinal Degenerative Diseases: Piezo1,” BioMed Research International 2021 (2021): 6645193. 33575334 10.1155/2021/6645193 PMC7857891

[29] S. Shi , X. J. Kang , Z. Zhou , et al., “Excessive Mechanical Stress‐Induced Intervertebral Disc Degeneration Is Related to Piezo1 Overexpression Triggering the Imbalance of Autophagy/Apoptosis in Human Nucleus Pulpous,” Arthritis Research & Therapy 24 (2022): 119. 35606793 10.1186/s13075-022-02804-y PMC9125856

[30] B. Wang , W. Ke , K. Wang , et al., “Mechanosensitive Ion Channel Piezo1 Activated by Matrix Stiffness Regulates Oxidative Stress‐Induced Senescence and Apoptosis in Human Intervertebral Disc Degeneration,” Oxidative Medicine and Cellular Longevity 2021, no. 1 (2021): 8884922. 33628392 10.1155/2021/8884922 PMC7889339

[31] R. Nakamichi , Y. Ito , M. Inui , et al., “Mohawk Promotes the Maintenance and Regeneration of the Outer Annulus Fibrosus of Intervertebral Discs,” Nature Communications 7 (2016): 12503. 10.1038/ncomms12503 PMC4990710 27527664

[32] O. M. Torre , V. Mroz , A. R. M. Benitez , et al., “Neonatal Annulus Fibrosus Regeneration Occurs via Recruitment and Proliferation of Scleraxis‐Lineage Cells,” npj Regenerative Medicine 4 (2019): 23. 31885875 10.1038/s41536-019-0085-4 PMC6925137

[33] B. M. Minogue , S. M. Richardson , L. A. Zeef , et al., “Transcriptional Profiling of Bovine Intervertebral Disc Cells: Implications for Identification of Normal and Degenerate Human Intervertebral Disc Cell Phenotypes,” Arthritis Research & Therapy 12 (2010): 1–20. 10.1186/ar2929 PMC2875656 20149220

[34] R. Nakamichi , S. Ma , T. Nonoyama , et al., “The Mechanosensitive Ion Channel piezo1 Is Expressed in Tendons and Regulates Physical Performance,” Science Translational Medicine 14 (2022): eabj5557. 35648809 10.1126/scitranslmed.abj5557

[35] C. Liu , X. Gao , J. Lou , et al., “Aberrant Mechanical Loading Induces Annulus Fibrosus Cells Apoptosis in Intervertebral Disc Degeneration via Mechanosensitive Ion Channel Piezo1,” Arthritis Research & Therapy 25 (2023): 117. 37420255 10.1186/s13075-023-03093-9 PMC10327399

[36] Y. Pan , L. Z. Shi , C. W. Yoon , et al., “Mechanosensor piezo1 Mediates Bimodal Patterns of Intracellular Calcium and FAK Signaling,” EMBO Journal 41 (2022): e111799. 35844093 10.15252/embj.2022111799 PMC9433934

[37] I. A. Stokes , “Surface Strain on Human Intervertebral Discs,” Journal of Orthopaedic Research 5 (1987): 348–355. 3625358 10.1002/jor.1100050306

[38] Z. Kazezian , R. Gawri , L. Haglund , et al., “Gene Expression Profiling Identifies Interferon Signalling Molecules and IGFBP3 in Human Degenerative Annulus Fibrosus,” Scientific Reports 5 (2015): 15662. 26489762 10.1038/srep15662 PMC4614807

[39] A. Cho , Y. Tang , J. Davila , et al., “Calcineurin Signaling Regulates Neural Induction Through Antagonizing the Bmp Pathway,” Neuron 82 (2014): 109–124. 24698271 10.1016/j.neuron.2014.02.015 PMC4011666

[40] S. Sangadala , E. J. Devereaux , S. M. Presciutti , et al., “Fk506 Induces Ligand‐Independent Activation of the Bone Morphogenetic Protein Pathway and Osteogenesis,” International Journal of Molecular Sciences 20 (2019): 1900. 30999619 10.3390/ijms20081900 PMC6515024

[41] F. Peng , M. Sun , X. Jing , et al., “Piezo1 Promotes Intervertebral Disc Degeneration Through the ca(2+)/F‐Actin/Yap Signaling Axis,” Molecular Medicine (Cambridge, Mass) 31 (2025): 90. 40057686 10.1186/s10020-025-01147-z PMC11889814

[42] Z. Lin , G. Xu , X. Lu , et al., “Piezo1 Exacerbates Inflammation‐Induced Cartilaginous Endplate Degeneration by Activating Mitochondrial Fission via the Ca(2+)/CaMKII/Drp1 Axis,” Aging Cell 24 (2025): e14440. 39610146 10.1111/acel.14440 PMC11984661

[43] C. Liu , X. Gao , J. Lou , et al., “Aberrant Mechanical Loading Induces Annulus Fibrosus Cells Apoptosis in Intervertebral Disc Degeneration via Mechanosensitive Ion Channel Piezo1,” Arthritis Research & Therapy 25 (2023): 117. 37420255 10.1186/s13075-023-03093-9 PMC10327399

[44] G. D. O'Connell , E. J. Vresilovic , and D. M. Elliott , “Human Intervertebral Disc Internal Strain in Compression: The Effect of Disc Region, Loading Position, and Degeneration,” Journal of Orthopaedic Research 29 (2011): 547–555. 21337394 10.1002/jor.21232 PMC3428014

[45] S. Sen , N. T. Jacobs , J. I. Boxberger , and D. M. Elliott , “Human Annulus Fibrosus Dynamic Tensile Modulus Increases With Degeneration,” Mechanics of Materials 44 (2012): 93–98. 22247579 10.1016/j.mechmat.2011.07.016 PMC3254102

[46] B. V. Fearing , P. A. Hernandez , L. A. Setton , and N. O. Chahine , “Mechanotransduction and Cell Biomechanics of the Intervertebral Disc,” JOR Spine 1 (2018): e1026. 30569032 10.1002/jsp2.1026 PMC6296470

[47] F. Rannou , P. Richette , M. Benallaoua , et al., “Cyclic Tensile Stretch Modulates Proteoglycan Production by Intervertebral Disc Annulus Fibrosus Cells Through Production of Nitrite Oxide,” Journal of Cellular Biochemistry 90 (2003): 148–157. 12938164 10.1002/jcb.10608

[48] H. Cho , A. Seth , J. Warmbold , et al., “Aging Affects Response to Cyclic Tensile Stretch: Paradigm for Intervertebral Disc Degeneration,” European Cells & Materials 22 (2011): 137–145, discussion 136–145. 21932191 10.22203/ecm.v022a11

[49] G. W. D. Easson , A. Savadipour , A. Anandarajah , et al., “Modulation of trpv4 Protects Against Degeneration Induced by Sustained Loading and Promotes Matrix Synthesis in the Intervertebral Disc,” FASEB Journal 37 (2023): e22714. 36583692 10.1096/fj.202201388R PMC10291737

[50] M. K. Mark Kim , M. Lawrence , D. Quinonez , C. Brooks , R. Ramachandran , and C. A. Séguin , “Transient Receptor Potential Vanilloid 4 Regulates Extracellular Matrix Composition and Mediates Load‐Induced Intervertebral Disc Degeneration in a Mouse Model,” Osteoarthritis and Cartilage 32 (2024): 881–894. 38604493 10.1016/j.joca.2024.04.001

[51] A. Savadipour , D. Palmer , E. V. Ely , et al., “The Role of Piezo Ion Channels in the Musculoskeletal System,” American Journal of Physiology. Cell Physiology 324 (2023): C728–C740. 36717101 10.1152/ajpcell.00544.2022 PMC10027092

[52] L. Wang , X. You , S. Lotinun , et al., “Mechanical Sensing Protein piezo1 Regulates Bone Homeostasis via Osteoblast‐Osteoclast Crosstalk,” Nature Communications 11 (2020): 282. 10.1038/s41467-019-14146-6 PMC6962448 31941964

[53] X. Ren , B. Li , C. Xu , et al., “High Expression of piezo1 Induces Senescence in Chondrocytes Through Calcium Ions Accumulation,” Biochemical and Biophysical Research Communications 607 (2022): 138–145. 35367826 10.1016/j.bbrc.2022.03.119

[54] A. Bernareggi , A. Bosutti , G. Massaria , et al., “The State of the Art of piezo1 Channels in Skeletal Muscle Regeneration,” International Journal of Molecular Sciences 23 (2022): 6616. 35743058 10.3390/ijms23126616 PMC9224226

[55] A. Sugimoto , A. Miyazaki , K. Kawarabayashi , et al., “Piezo Type Mechanosensitive Ion Channel Component 1 Functions as a Regulator of the Cell Fate Determination of Mesenchymal Stem Cells,” Scientific Reports 7 (2017): 17696. 29255201 10.1038/s41598-017-18089-0 PMC5735093

[56] G. Chen , C. Deng , and Y. P. Li , “TGF‐β and Bmp Signaling in Osteoblast Differentiation and Bone Formation,” International Journal of Biological Sciences 8 (2012): 272–288. 22298955 10.7150/ijbs.2929 PMC3269610

