# Supplementary figures and images for: Mechanosensitive Ion Channel PIEZO1 Suppresses BMP2‐Induced Ossification of the Annulus Fibrosus Cells
Source: JOR Spine. 2026 Mar 3;9(1):e70168. doi: 10.1002/jsp2.70168 (PMC12954436; doi:10.1002/jsp2.70168)

**A**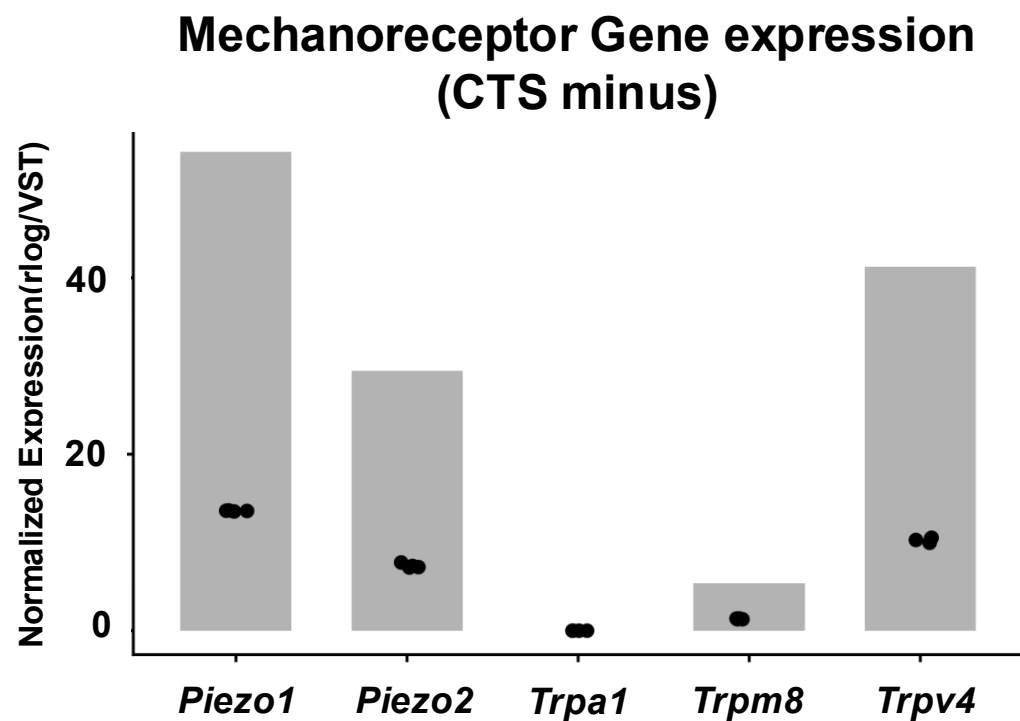**B**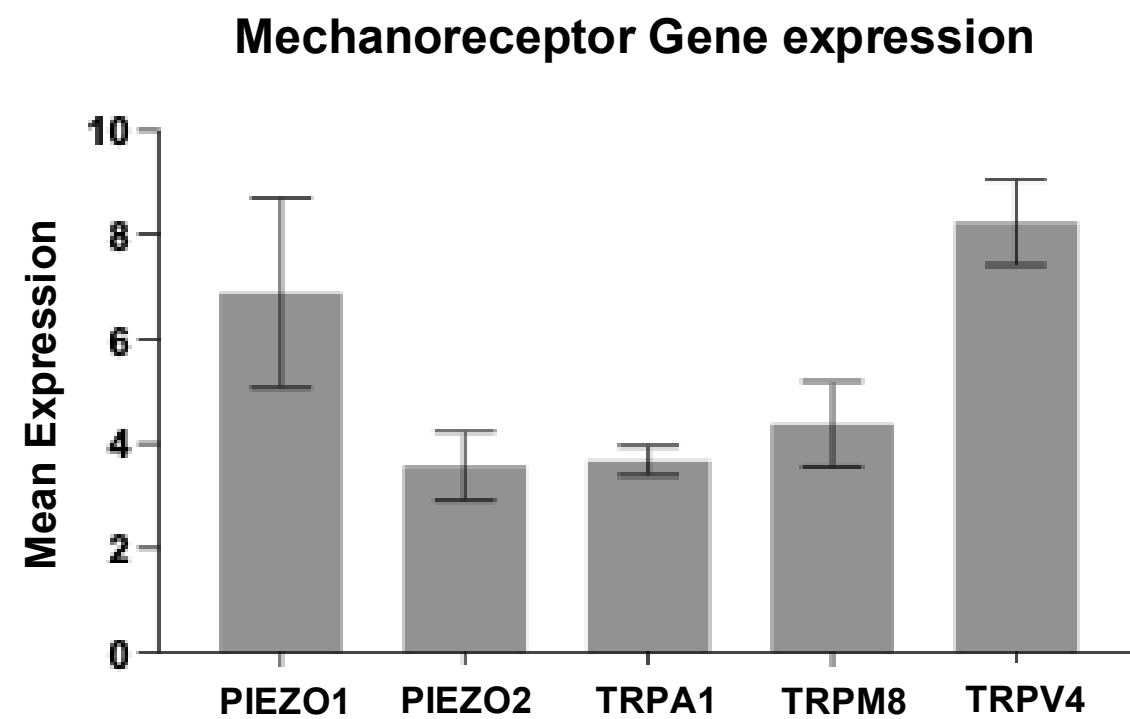

Supplement: Supplementary file 1 — Figure S1: Basal expression of mechanoreceptor genes in the AF tissue. (A) Baseline expression levels of representative mechanoreceptor genes in rat AF cells, assessed by RNA‐seq using samples from the CTS‐minus (control) group. Multiple mechanoreceptor genes were detectably expressed in rat AF cells, among which Piezo1 and Trpv4—mechanoreceptors previously reported to play functional roles in musculoskeletal tissues—exhibited relatively higher basal expression levels compared with other candidates. (B) Gene expression profiling of mechanoreceptor genes in human AF tissue using publicly available microarray data (GEO accession number: GSE70362). Consistent with the rat data, PIEZO1 and TRPV4 showed higher expression levels than other mechanoreceptor genes in normal human intervertebral discs, supporting their potential involvement in mechanotransduction in AF tissue. [file JSP2-9-e70168-s004.pdf]

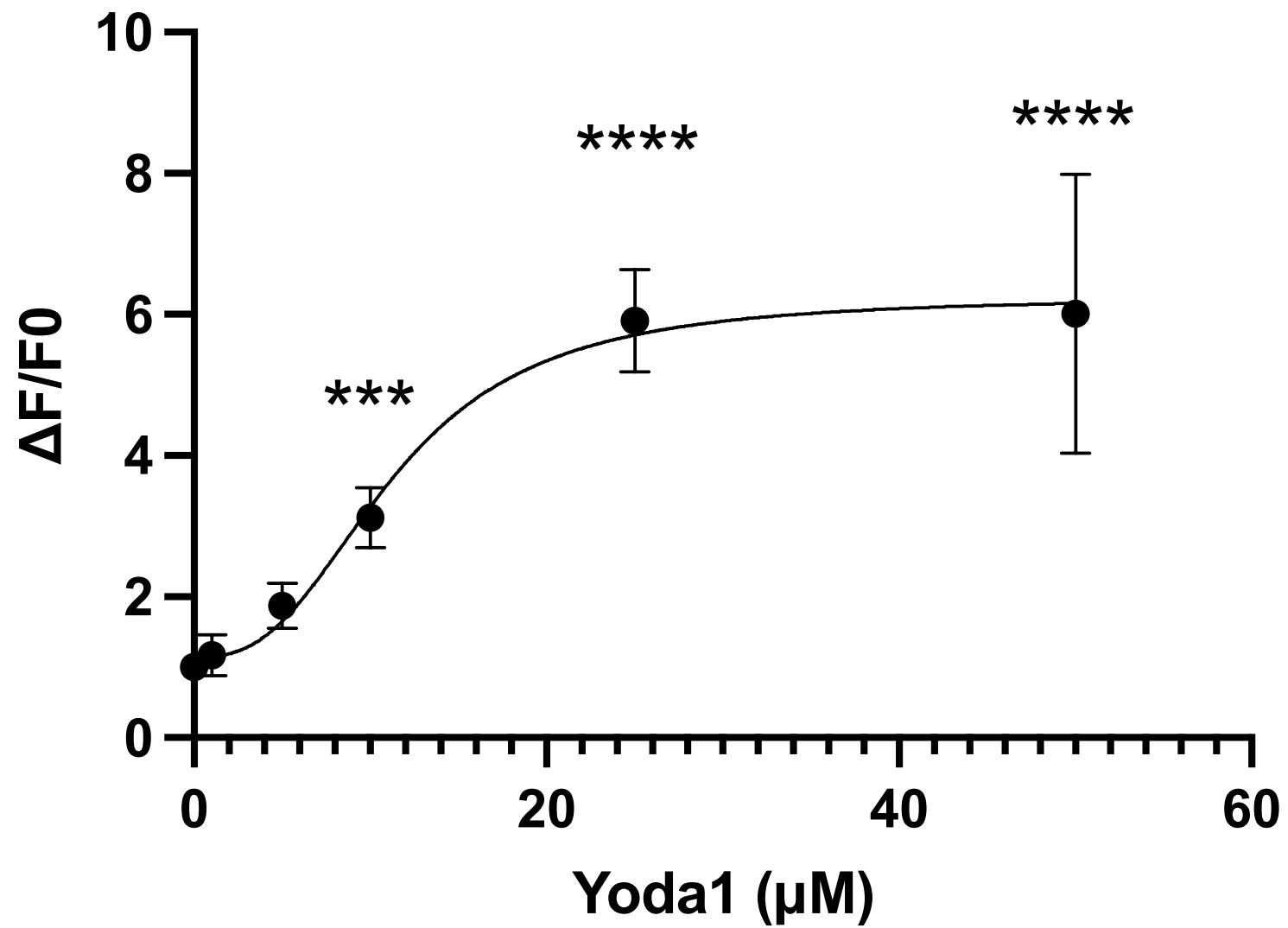

Supplement: Supplementary file 2 — Figure S2: Intracellular calcium influx in AF cells following Piezo1 activation by Yoda1. AF cells were treated with increasing concentrations of Yoda1 (1, 5, 10, 25, and 50 μM), and intracellular calcium influx was measured using a calcium‐sensitive fluorescent dye. Calcium influx significantly increased at ≥ 10 μM, indicating the dose‐dependent activation of Piezo1 channels. [file JSP2-9-e70168-s013.pdf]

**A**

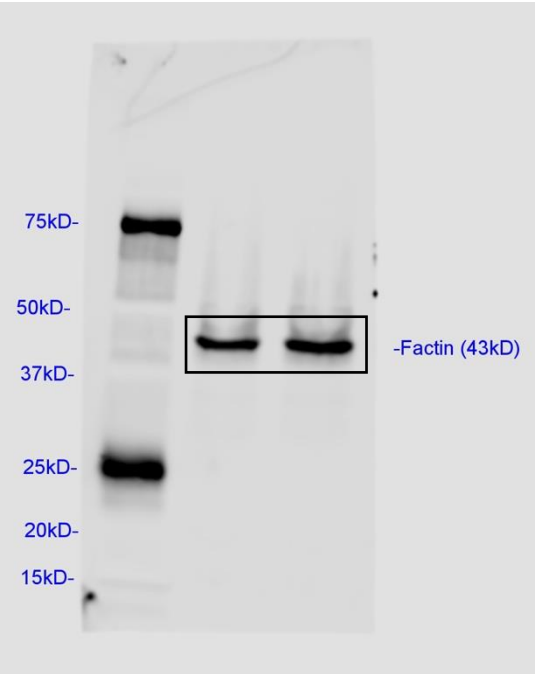

**B**

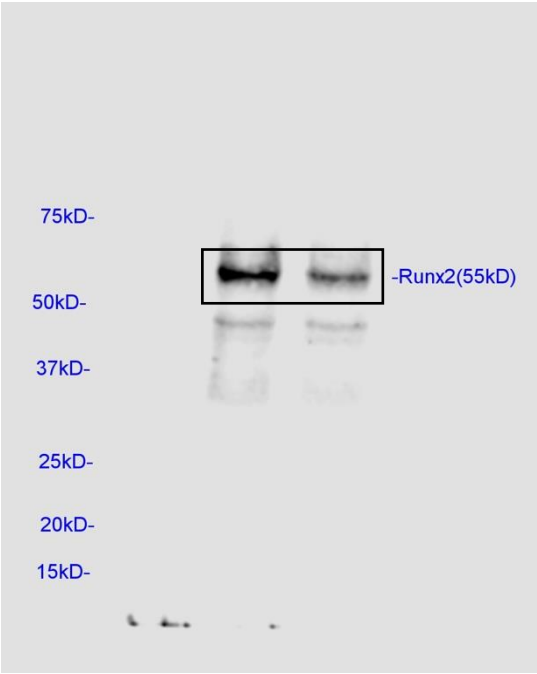

**C**

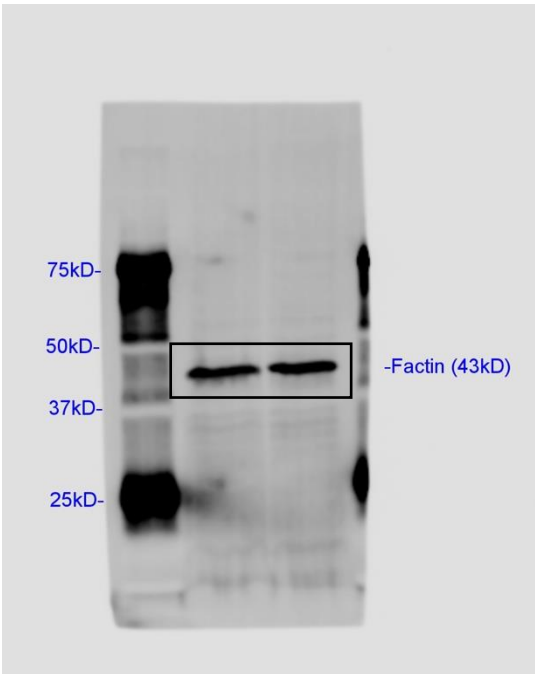

**D**

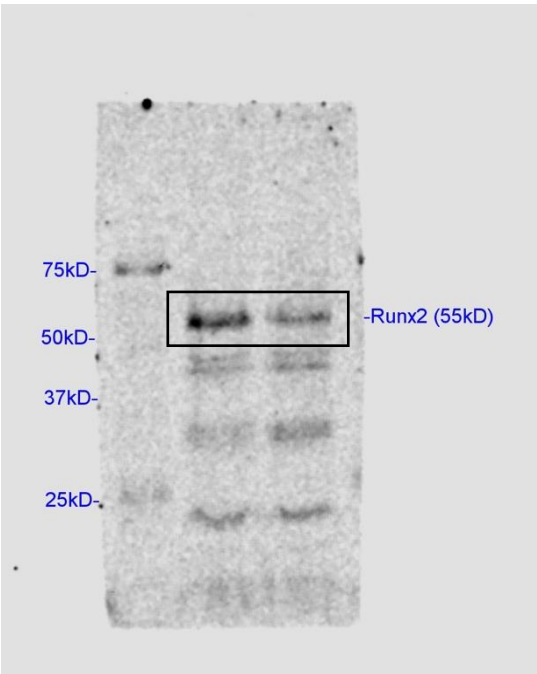

**E**

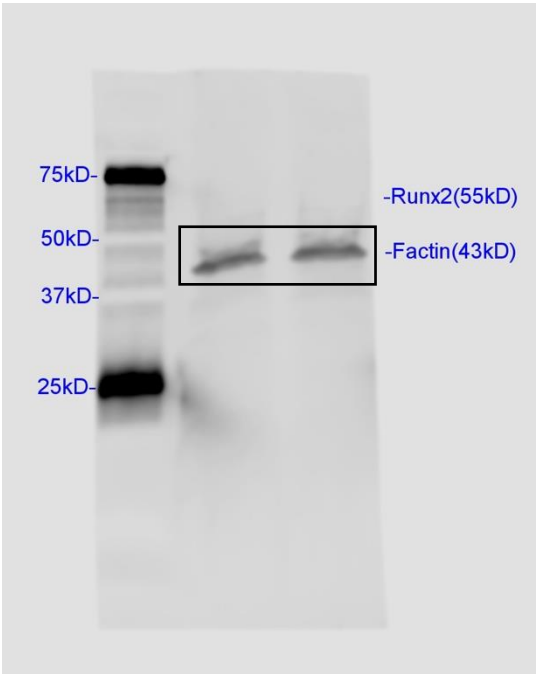

**F**

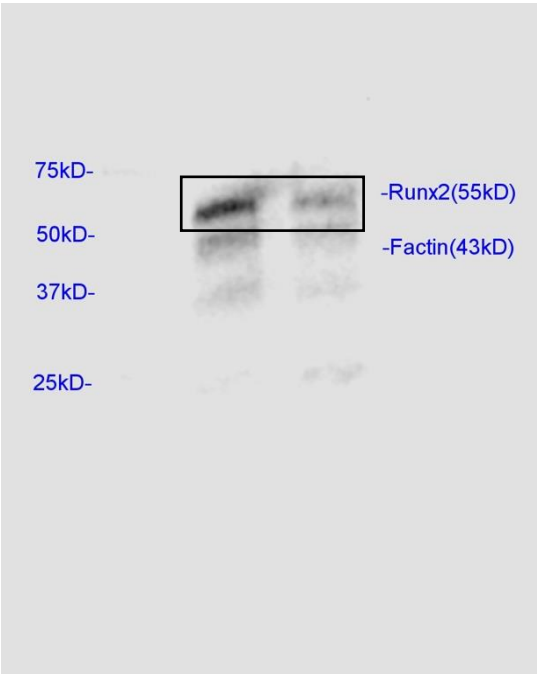

Supplement: Supplementary file 3 — Figure S3: Full‐length images of the Western blotting shown in Figures 4B and 5C,E. Original, uncropped Western blotting images corresponding to the cropped panels in Figure 4B (rat AF cells), 5C and E (human AF cells) are presented. These images validate the reduction of RUNX2 protein expression after treatment with the Piezo1 agonist Yoda1. (A, B): rat, (C, D): human, Pfirrmann grade 1, (E, F): human, Pfirrmann grade 5. [file JSP2-9-e70168-s014.pdf]

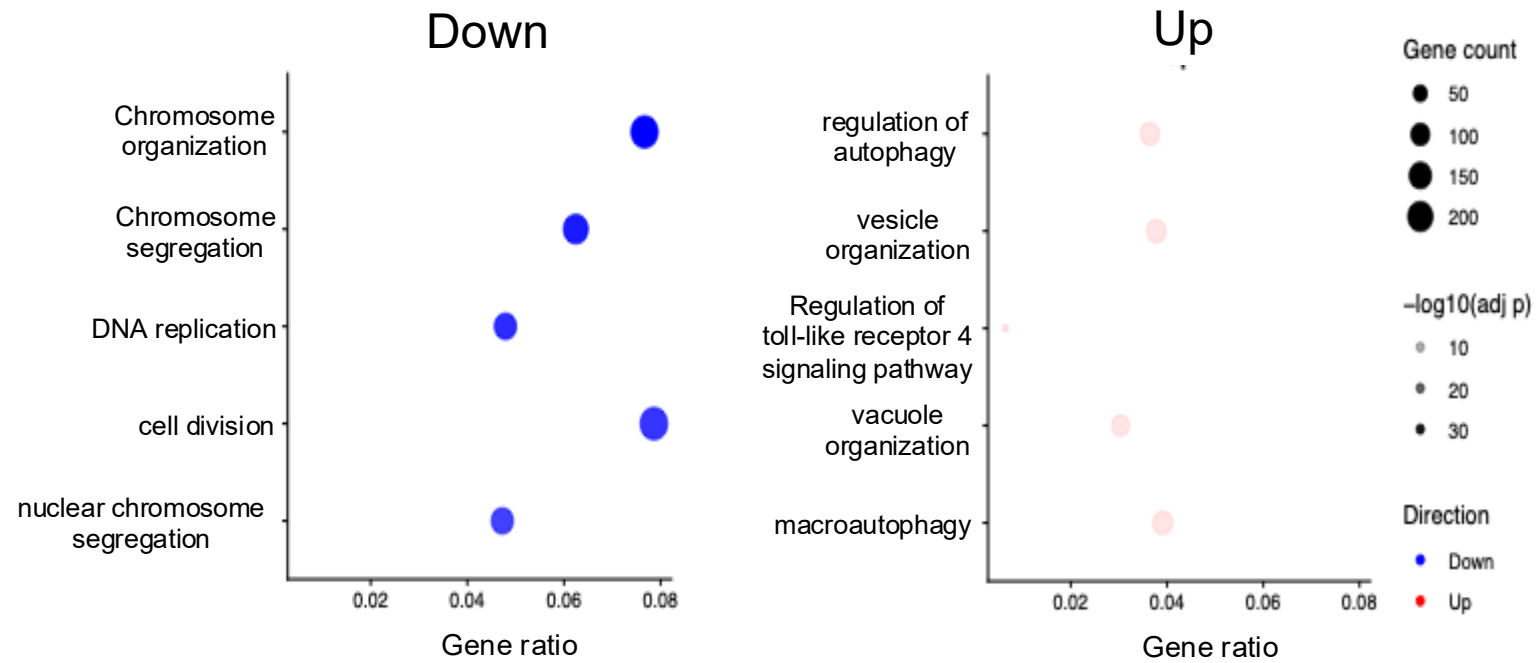

Supplement: Supplementary file 4 — Figure S4: GO analysis of Yoda1‐treated AF cells. GO enrichment analysis revealed that genes downregulated by Piezo1 activation were predominantly associated with cell cycle‐ and chromosome‐related processes, including chromosome organization and DNA replication. In contrast, upregulated genes were enriched in autophagy‐ and vesicle‐related pathways, such as regulation of autophagy and vesicle organization. These findings suggest that Piezo1 activation induces a transcriptional shift from proliferative programs toward intracellular regulatory and signaling processes in AF cells. [file JSP2-9-e70168-s010.pdf]

**A**

Downregulated overlap

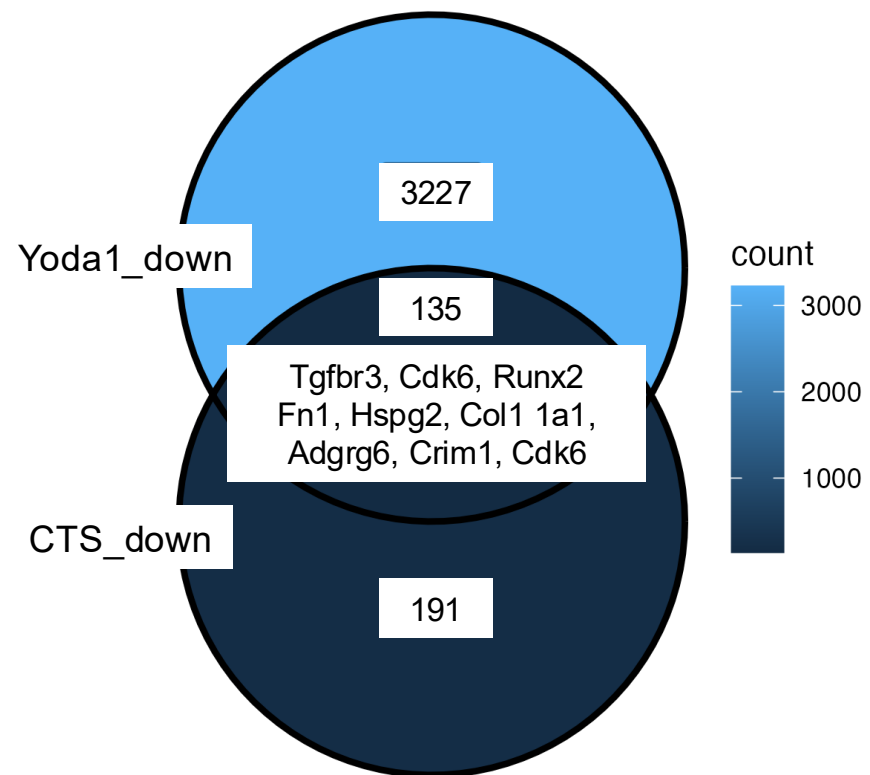**B**

GSEA: regulation of ossification (GO:0030278)

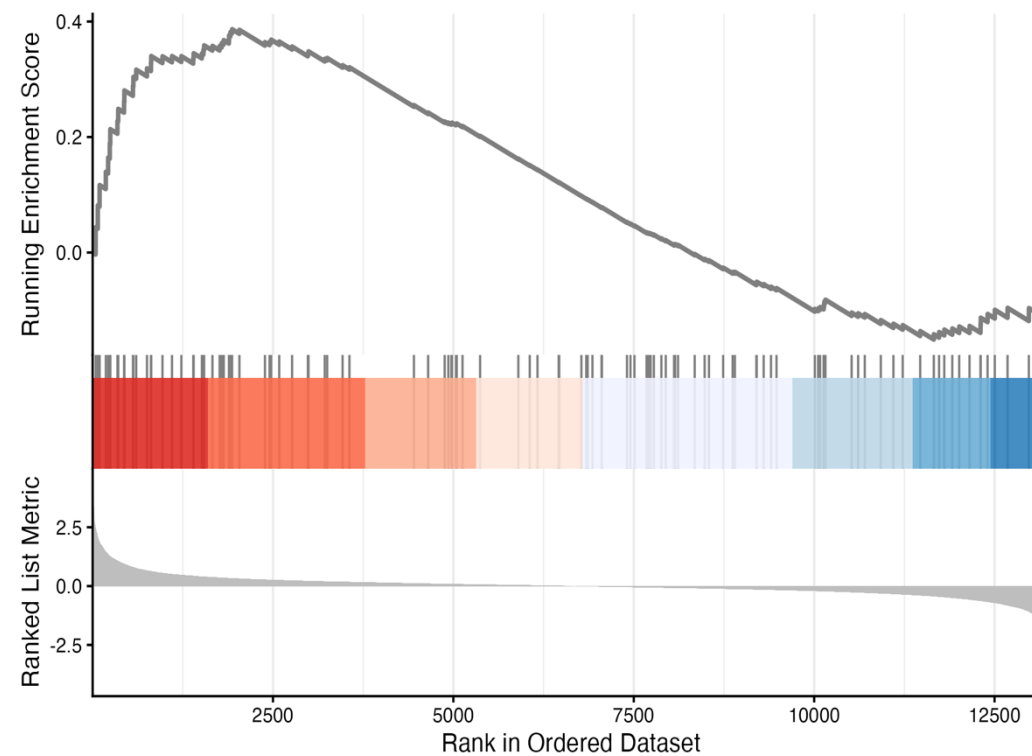

Supplement: Supplementary file 5 — Figure S5: Comparison of transcriptomic responses to moderate CTS and pharmacological Piezo1 activation in AF cells. (A) Venn diagram illustrating the overlap of genes downregulated by moderate CTS and Yoda1 treatment. A total of 135 genes were commonly downregulated under both conditions. Among these, nine genes—including Runx2—were annotated to ossification‐related GO terms. (B) GSEA performed using the ranked gene list demonstrated significant enrichment of the GO term “regulation of ossification” among genes commonly downregulated by CTS and Yoda1 treatment. Together, these data indicate that mechanical stimulation and pharmacological activation of Piezo1 elicit partially overlapping transcriptional programs associated with suppression of ossification‐related pathways in AF cells. [file JSP2-9-e70168-s003.pdf]

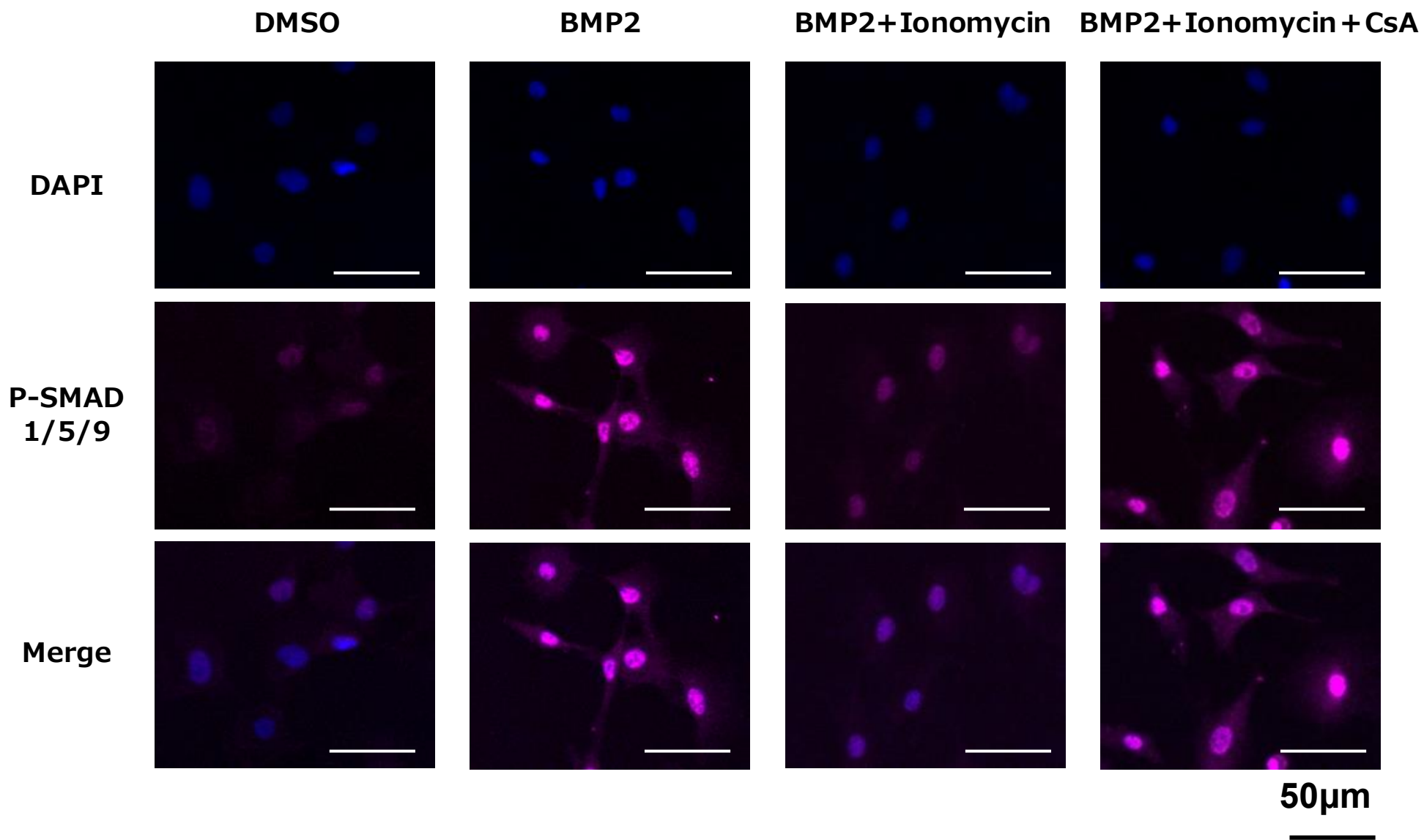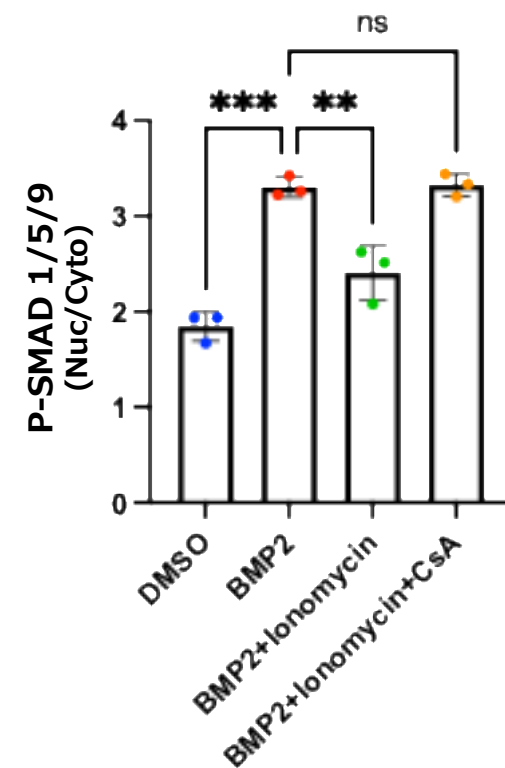

Supplement: Supplementary file 6 — Figure S6: Immunocytochemistry following Ionomycin treatment. Ionomycin was used as a pharmacological calcineurin activator. Under BMP2 co‐treatment, ionomycin reduced nuclear translocation of p‐Smad1/5/9, indicating calcineurin‐dependent suppression of BMP‐Smad signaling. [file JSP2-9-e70168-s002.pdf]

**A****FAST staining**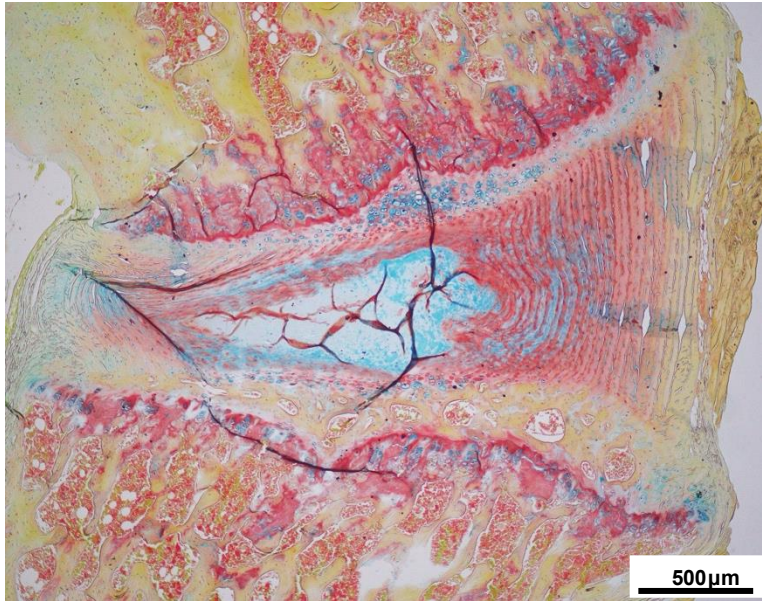**B****IHC (PIEZO1),  $\times 40$** 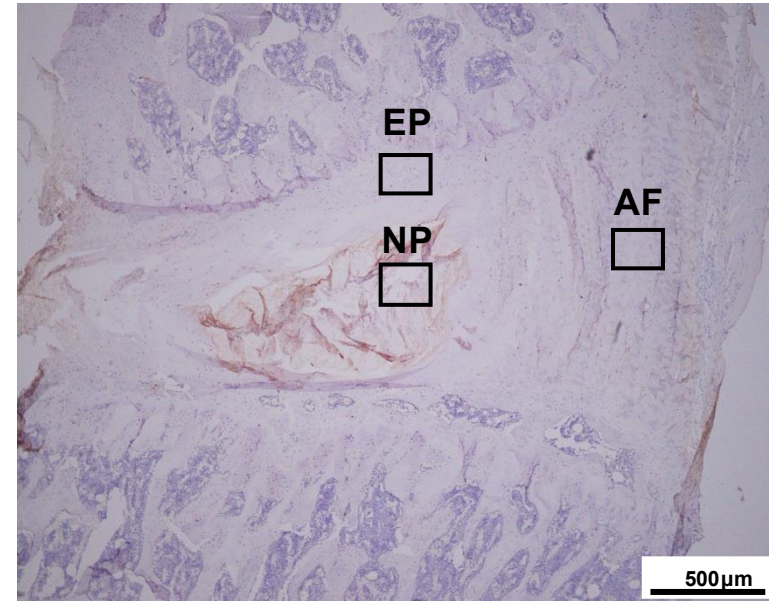**C****IHC (PIEZO1),  $\times 400$** **NP**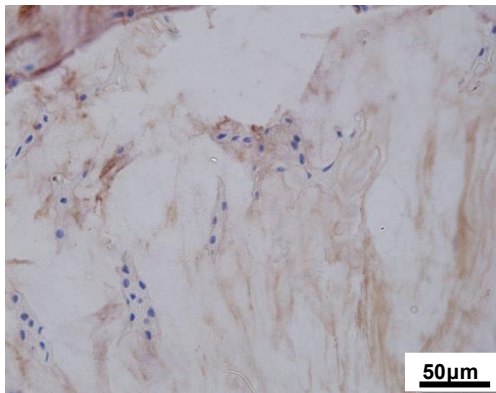**AF**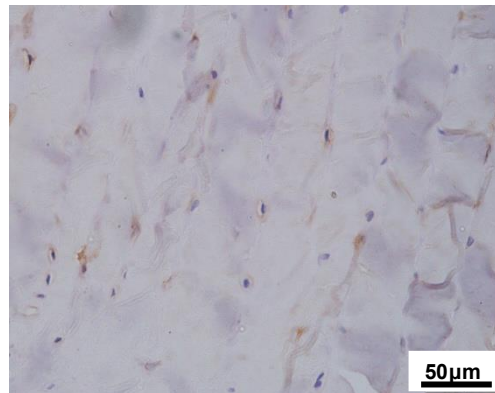**EP**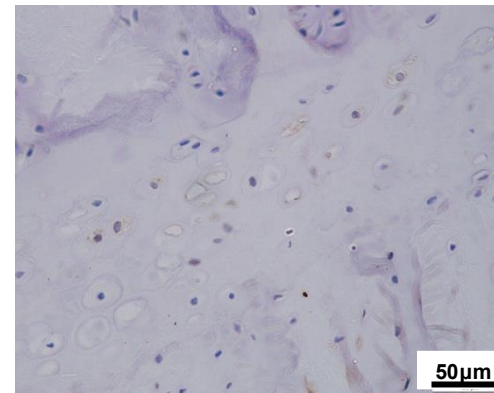**D****PIEZO1**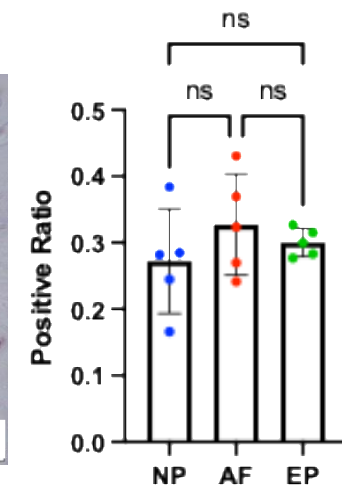

Supplement: Supplementary file 7 — Figure S7: Immunohistochemistry of rat IVD. (A) FAST staining of rat IVD. (B) Immunohistochemistry for PIEZO1. PIEZO1 was expressed throughout the AF, NP, and CEP, with no appreciable difference in expression levels among these regions. [file JSP2-9-e70168-s009.pdf]

***Bmp2***  
**(Rat)**

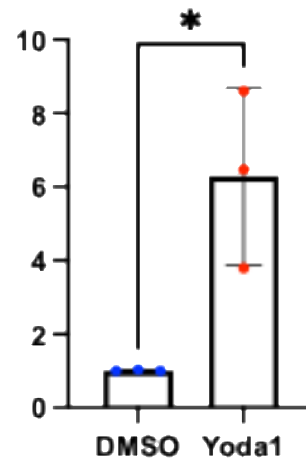

**BMP2**  
**(Pfirrman 1)**

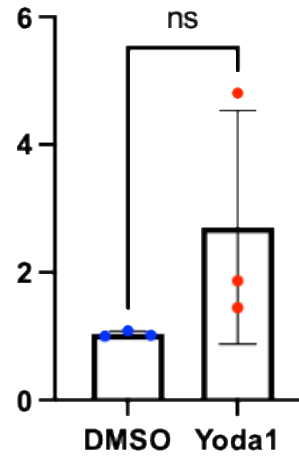

**BMP2**  
**(Pfirrman 5)**

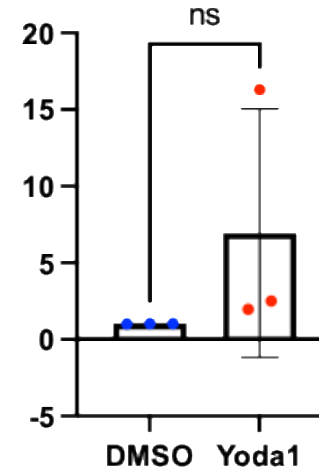

Supplement: Supplementary file 8 — Figure S8: Bmp2 mRNA expression following Yoda1 treatment. Yoda1 treatment tended to increase BMP2 mRNA expression in both rat and human AF cells. [file JSP2-9-e70168-s007.pdf]

***Piezo1 (Low)***

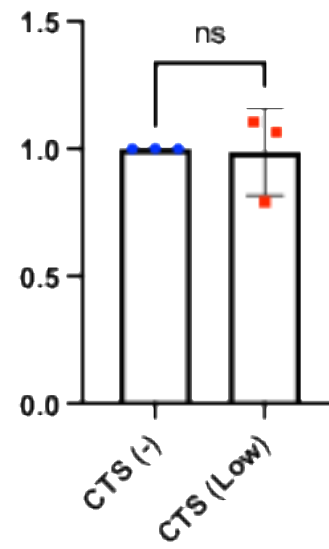

***Piezo1 (High)***

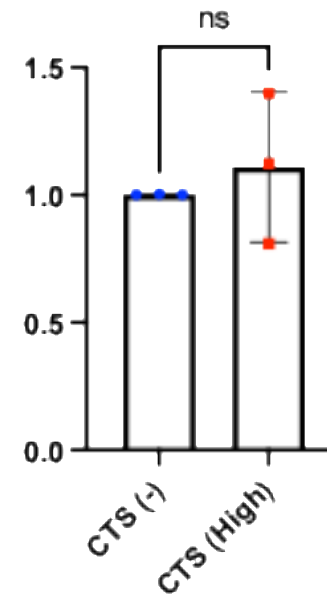

Supplement: Supplementary file 9 — Figure S9: Piezo1 mRNA expression following CTS. Piezo1 expression was not significantly altered under either low‐ and high‐intensity CTS. [file JSP2-9-e70168-s005.pdf]
