# Supplementary material for: Mechanosensitive Ion Channel PIEZO1 Suppresses BMP2‐Induced Ossification of the Annulus Fibrosus Cells
Source: JOR Spine. 2026 Mar 3;9(1):e70168. doi: 10.1002/jsp2.70168 (PMC12954436; doi:10.1002/jsp2.70168)
Supplement: Supplementary file 10 — Table S1: Human sample metadata. Table S2: Primer list of rats. Table S3:. Primer list of humans. [file JSP2-9-e70168-s012.docx]

**Supplementary Tables**

**Supplementary Table 1: Human sample metadata**

|  | Age | Sex | level | Pfirrmann grade | diagnosis | operation |
| --- | --- | --- | --- | --- | --- | --- |
| 1 | 77 | M | L4/5 | 5 | Lumbar canal stenosis | Oblique lateral body interfusion |
| 2 | 78 | F | L3/4 | 5 | Lumbar canal stenosis | Corpectomy |
| 3 | 71 | F | L2/3 | 5 | Lumbar canal stenosis | Oblique lateral body interfusion |
| 4 | 16 | M | L1/2 | 1 | Adolescent idiopathic scoliosis | Anterior collection and fusion |
| 5 | 16 | M | L2/3 | 1 | Adolescent idiopathic scoliosis | Anterior collection and fusion |
| 6 | 16 | F | L1/2 | 1 | Adolescent idiopathic scoliosis | Anterior collection and fusion |

**Supplementary Table 2: Primer list of rats**

| Gene | Forward primer | Reverse primer |
| --- | --- | --- |
| *Gapdh* | 5′-GGCAAGTTCAATGGCACAGT-3′ | 5′-TGGTGAAGACGCCAGTAGACTC-3′ |
| *Runx2* | 5′-GGGAACCAAGAAGGCACAGA-3′ | 5′-GGATGAGGAATGCGCCCTAA-3′ |
| *Osx* | 5′-CTAGCCTCCCCAGGAAGAAGC-3′ | 5′-CCAGGGCTGTTGAGTCTCG-3′ |
| *Alp* | 5′-GCACAACATCAAGGACATCG-3′ | 5′-TCAGTTCTGTTCTTGGGGTACAT-3′ |
| *OCN* | 5′-AGGACCCTCTCTCTGCTCAC-3′ | 5′-AACGGTGGTGCCATAGATGC-3′ |
| *Il-6* | 5′-TCTTGGAAATGAGAAAAGAGTTGTG-3′ | 5′-ACGGAACTCCAGAAGACCAGA-3′ |
| *Cox2* | 5′-CTCAGCCATGCAGCAAATCC-3′ | 5′-GGGTGGGCTTCAGCAGTAAT-3′ |
| *Piezo1* | 5′-AGGCTGGACCAGGACCTACT-3′ | 5′-TGCGTTGTCCGATGACATTC-3′ |
| *Piezo2* | 5′-GTTGCCCATCCTGATCTTCCT-3′ | 5′-TCACTACAATCGCCACCTCA-3′ |
| *Trpv4* | 5′-ACAGCAAGATCGAGAACCGC-3′ | 5′-CTTACGCCACTTGTCCCTCA-3′ |
| *BMP2* | 5′-GCTTCCATCACGAAGAAGCCA-3′ | 5′-ATTGAAGAAGAAGCGTCGGGA-3′ |

**Supplementary Table 3: Primer list of humans**

| Gene | Forward primer | Reverse primer | |
| --- | --- | --- | --- |
| *GAPDH* | 5′-ACCATCTTCCAGGAGCGAGA-3′ | 5′-GACTCCACGACGTACTCAGC-3′ | |
| *RUNX2* | 5′-GGCGCATTTCAGATGATGACA-3′ | 5′-CCTGCCTGGCTCTTCTTACTG-3′ | |
| *OSX* | 5′-ATCCAGCCCCCTTTACAAGC-3′ | 5′-TAGCATAGCCTGAGGTGGGT-3′ | |
| *ALP* | 5′-GACCAAGCGCAAGAGACACT-3′ | 5′-GGAGACACCCATCCCATCTC-3′ |  |
| *BMP2* | 5′-GCAGCTTCCACCATGAAGAATC-3′ | 5′-TCCTCCGTGGGGATAGAACT-3′ |  |
