# Supplementary material for: Mechanosensitive Ion Channel PIEZO1 Suppresses BMP2‐Induced Ossification of the Annulus Fibrosus Cells
Source: JOR Spine. 2026 Mar 3;9(1):e70168. doi: 10.1002/jsp2.70168 (PMC12954436; doi:10.1002/jsp2.70168)
Supplement: Supplementary file 15 — Data S1: jsp270168‐sup‐0015‐supinfo.docx. [file JSP2-9-e70168-s001.docx]

**Supplementary Materials and Methods**

**Rat AF primary cell isolation**

Primary AF cells were isolated using the following method. Ten-week-old male Wistar rats were euthanized via CO_2_ inhalation. The intervertebral discs from L1–S1 levels were immediately extracted under sterile conditions. The NP and inner AF were removed by using a dermal biopsy punch (Kai Medical, Tokyo, Japan, Cat. #BP-10F), isolating only the outer AF.

The extracted tissues were placed in cooled phosphate-buffered saline (PBS; Nacalai Tesque, Kyoto, Japan, Cat. #14249-95) supplemented with 2% penicillin–streptomycin–amphotericin B (PSA) and washed once to remove blood and impurities. Under clean ventilation conditions, the washed AF tissues were finely minced into approximately 1 mm^3^ fragments and subsequently washed twice with PBS. Subsequently, for enzymatic digestion, the minced tissue was transferred in a 15 mL tube and incubated at 37°C in a shaking incubator (300 rpm) with 0.4% Pronase (Roche, Basel, Switzerland, Cat. #10165921001) for 1 h, followed by 0.025% collagenase P (Roche, Cat. #11213857001) for 3 h, with gentle agitation at regular intervals. After enzymatic digestion, the cell suspension was filtered through a 70-µm cell strainer (AS ONE, Osaka, Japan, Cat. #VCS-70) to remove debris. Subsequently, the filtered suspension was centrifuged (1500 rpm, 5 min), and the resulting cell pellet was resuspended in Dulbecco’s Modified Eagle Medium (DMEM; Nacalai Tesque, Cat. #08456-65) supplemented with 10% fetal bovine serum (FBS; HyClone Laboratories, Logan, UT, Cat. #SH30071.03) and 1% PSA. This washing step was repeated twice. Subsequently, the isolated cells were seeded onto 10-cm type I collagen-coated dishes (AGC Techno Glass, Shizuoka, Japan, Cat. #4020-010).

The cells were cultured in an incubator maintained at 37°C with a humidified atmosphere of 5% CO_2_, and the medium was replaced with fresh medium every 2 days. The cells were collected when they reached 80%–90% confluence and cryopreserved at −80°C in aliquots containing 5.0 × 10⁵ cells. All experiments were conducted using only Passage 0 cells, and at least three different cell isolations collected at different time points were used.

**Human AF primary cell isolation**

Human AF cells were isolated from intervertebral discs (IVDs) obtained from two distinct patient groups. First, non-degenerated IVD tissues (Pfirrmann grade 1) were collected from adolescent patients (mean age: 16.0 years) undergoing corrective surgery for adolescent idiopathic scoliosis (AIS). Second, severely degenerated IVD tissues (Pfirrmann grade 5) were obtained from older adults (mean age: 75.3 years) undergoing lumbar surgeries, including lateral lumbar interbody fusion or corpectomy, at levels L1–L5. Patient demographic information is summarized in Supplementary Table 1. The Ethics Committee of Okayama University approved all procedures (Approval No. 2212-021), and all patients provided informed consent preoperatively. As with rat AF cell isolation, the harvested AF tissues were washed and minced. The tissues were digested with **0.4% Pronase (Roche, Cat. #10165921001) for 1 h**, followed by **0.25% collagenase II (Sigma Aldrich, St. Louis, MO, Cat. #C2-22) + 0.01% hyaluronidase (Sigma Aldrich, Cat. #H2251) for 4 h**. For all subsequent experiments, **Passage 1–2 cells** were used, and at least three independent cell lines derived from different patients were utilized.

**Cyclic tensile strain**

To enhance cell adhesion, flexible **stretch chambers** (2 × 2 cm) (Menicon Life Sciences, Nagoya, Japan, Cat. #SC4Dea) made of **polydimethylsiloxane (PDMS)** were coated with **collagen type I** (Corning, NY, Cat. #354236). The cells were seeded at **8.0 × 10^4^** and cultured at **37°C** in a **5% CO_2_** incubator for **48 h.** The cells were then cultured under low-serum conditions (2% FBS) for 12h before being subjected to CTS using ShellPa Pro (Menicon Life Sciences). Two different mechanical strains were applied: low- (strain: 2%, frequency: 1 Hz, duration: 12 h) and high-strain stimulation (strain: 12%, frequency: 1 Hz, duration: 12 h). Control cells were subjected to identical conditions but without CTS exposure.

**Microarray analysis**

The microarray dataset was obtained from the GEO; accession number **GSE70362)**. The dataset includes expression profiles from **eight normal samples** of AF tissue from intervertebral discs. The data were generated using the Affymetrix Human Genome U133 Plus 2.0 Array platform. The raw CEL files were processed using the robust multi-array average method, including background correction, quantile normalization, and log2 transformation. The probe set annotation was updated using the Brainarray custom chip definition files to ensure accurate gene-level mapping. Mechanoreceptor genes were chosen based on their established roles in mechanical signal transduction. From this predefined list, only genes with detectable expression in the microarray dataset were selected for further analysis. Specifically, *PIEZO1*, *PIEZO2*, *TRPA1*, *TRPM8*, and *TRPV4* were analyzed because their corresponding probe sets exhibited expression above the background threshold.

**RNA interference**

All RNA interference (RNAi) experiments were performed using synthetic **small interfering RNA (siRNA)** reagents from **Dharmacon (Lafayette, CO)**. The following siRNA constructs were used: siControl: Cat. #D-001810-01-05, siPiezo1: Cat. #J-093854-10-0005, siPiezo2: Cat. #J-092136-09-0002, and siTrpv4: Cat. #J-095371-05-0002. **RNase-free water was used to prepare** stock solutions of **50 µM siRNA**. **Reverse transfection** was performed using **Lipofectamine RNAiMAX** (Thermo Fisher Scientific, Waltham, MA, Cat. #STEM00015) following the manufacturer’s instructions. To achieve a final siRNA concentration of **10 nM per well**, siRNA was diluted in **Opti-MEM** (Thermo Fisher Scientific, Cat. #31985062) and mixed with **Lipofectamine RNAiMAX** to form siRNA-Lipofectamine complexes before cell seeding. Using **RT-qPCR, knockdown efficiency** was assessed **48 h post-transfection**, confirming an efficiency of **≥80%**.

**Calcium assay**

Cells were seeded in **a 96-well plate** (AGC Techno Glass, Cat. #4860-010) and cultured overnight at **37°C in a 5% CO_2_ incubator**. The cells were stained with the calcium indicator dye **Fluo-8 NW** (AAT Bioquest, Pleasanton, CA, Cat. #36315) following the manufacturer’s instructions. Calcium responses were measured by recording intracellular calcium concentration changes after adding PIEZO1 agonist **Yoda1** (Selleck, Houston, TX, Cat. #S6678) with final concentrations of 1, 5, 10, 25, and 50μM. Fluorescence was measured using **FlexStation 3** (Molecular Devices, San Jose, CA), with an excitation wavelength and fluorescence emission of **490 and 525 nm, respectively**. The **maximum fluorescence change** was calculated by subtracting the baseline fluorescence intensity. The **maximum fluorescence intensity change (ΔF/F₀)** was determined using the control data as the reference.

**Real-time quantitative PCR (RT-qPCR)**

RNA was extracted using the **Direct-zol RNA Microprep Kit** (Zympo Research, Irvine, CA, Cat. #R2062) following the manufacturer’s instructions. RNA concentration and purity were measured using a **NanoDrop Lite** (Thermo Fisher Scientific). Subsequently, RNA was reverse-transcribed into complementary DNA using the **PrimeScript RT Master Mix** (Takara Bio, Shiga, Japan, Cat. #RR036A) with a **TProfessional TRIO 48 Thermocycler** (Analytik Jena, Jena, Germany). Real-time quantitative PCR (RT-qPCR) was performed using the **QuantStudio 1 Real-Time PCR System** (Thermo Fisher Scientific). The RT-qPCR reactions were performed using the **Brilliant III Ultra-Fast SYBR Green QPCR Master Mix** (Agilent Technologies, Santa Clara, CA, Cat. #600882). Target gene primers were designed using **NCBI Primer-BLAST**, and the sequences of the primers used are listed in Supplementary **Tables 1 and 2**. The **ΔΔCt method was used to calculate** relative gene expression levels, with normalization to the housekeeping gene **glyceraldehyde-3-phosphate dehydrogenase (Gapdh)**.

**RNA-sequencing (RNA-seq)**

For CTS experiments, AF cells were seeded at **8.0 × 10^4^** and cultured at **37°C** in a **5% CO_2_** incubator for **48 h.** After starvation with **DMEM containing 2% FBS** for **12 h,** cells were subjected to CTS (strain: 2%, frequency: 1 Hz, duration: 12 h), while non-stretched cells served as controls. For Yoda1 treatment AF cells were seeded at a density of **4.0 × 10^4^ cells per well** in a **24-well plate** (AGC Techno Glass, Cat. #4820-010) and cultured overnight at **37°C in a 5% CO_2_ incubator**. After confirming cell adhesion, **Yoda1 (10 μM)** was added to the experimental group, whereas **DMSO** was added to the control group. After 12 h of treatment, total RNA was extracted as described above. **RNA integrity** was assessed using **Tapestation 4150** (Agilent Technologies). Samples with an **RNA integrity number (RIN) ≥8.0** were used for RNA-sequencing analysis.

**RNA-seq** was performed by **AZENTA Life Sciences (Burlington, MA)** using four biological replicates per group (control and CTS groups, n = 4 each) and three biological replicates per group (DMSO and Yoda1 groups, n = 3 each). The mRNA was extracted using **poly(A) selection**, fragmented, and processed for library preparation using **the NEBNext Ultra II Directional RNA Library Prep Kit for Illumina** (New England BioLabs, Ipswich, MA, Cat. #E7760). The prepared libraries were sequenced on the **Illumina NovaSeq 6000** platform, generating **paired-end 2 × 150 bp reads**. The raw sequencing data underwent **quality checking and filtering** by AZENTA. Differentially expressed gene (DEG) analysis (padj < 0.1), Gene Ontology (**GO) analysis** and **Gene set enrichment analysis (GSEA)**were performed using **Rstudio (v2025.09.2+418)**.

**Western blotting**

Before protein extraction, the cells were washed twice with **ice-cold PBS containing a protease inhibitor** (Takara Bio, Cat. #ST0293). Nuclear proteins were extracted using the **EPIXTRACT Nuclear Protein Isolation Kit II** (Enzo Life Sciences, Farmingdale, NY, Cat. #ENZ-45015) following the manufacturer’s protocol. The protein concentration of the obtained extracts was quantified using the **Bradford Protein Assay Kit** (Takara Bio, Cat. #T9310A). Samples (**10 µg**) were prepared by mixing with **Laemmli Sample Buffer** (Bio-Rad, Hercules, CA, Cat. #1610747) supplemented with **2-mercaptoethanol** (Sigma Aldrich, Cat. #M3148) for **sodium dodecyl sulfate-polyacrylamide gel electrophoresis (SDS-PAGE)**. Each sample was loaded onto **Mini-Protean TGX gels** (Bio-Rad, Cat. #4561034) and electrophoresed at **100 V for 15 min, followed by 150 V for 1 h**. After electrophoresis, the proteins were transferred onto polyvinylidene difluoride (**PVDF) membranes** (Bio-Rad, Cat. #10026934) using the **Trans-Blot Turbo system** (Bio-Rad). Subsequently, the PVDF membranes were blocked with **Odyssey Blocking Buffer** (LI-COR Biosciences, Lincoln, NE, Cat. #92740000) at **room temperature for 1 h** and incubated with **primary antibodies** overnight at **4°C**. The primary antibodies used were as follows: Runx2 (1:1000 dilution, Rabbit Anti-Runx2, Cell Signaling Technology, Danvers, MA, Cat. #12556) and F-Actin (1:1000 dilution, Anti-Actin hFAB Rhodamine, Bio-Rad, Cat. #12004163). The next day, the membranes were washed with **PBS containing 0.1% Tween-20** and incubated with **IRDye goat anti-rabbit IgG** (LI-COR Biosciences, Lincoln, NE, Cat. #926-68071) as a **secondary antibody** for **1 h at room temperature**. **Immunoreactive proteins** were detected using the **Odyssey Fc Imaging System** (LI-COR Biosciences). Protein expression levels were normalized to the **housekeeping protein (F-Actin)**, and the **relative protein expression** of each sample was calculated. All experiments were performed in at least **three independent replicates**.

**Osteogenic differentiation**

The cells were seeded at a density of **4.0 × 10^4^ cells per well** in a **24-well plate** and cultured overnight at **37°C in a 5% CO_2_ incubator**. After confirming cell adhesion, **osteogenic differentiation** was induced by adding **recombinant human BMP2 (R&D Systems, Minneapolis, MN, Cat. #355-BEC-010)** every **72 h**. For the **control group**, an equivalent volume of **4 mM HCl** was added in place of BMP2. Osteogenic differentiation was assessed using **RT-qPCR** and **Alizarin Red staining after 2 and 3 weeks of culture, respectively**. **The administered drug concentrations are as follows: BMP2 (100 ng/mL), Yoda1 (10 μM), cyclosporin A (CsA; 100 nM, Fujifilm, Tokyo, Japan, Cat. #031-24931), and** Ionomycin (**1 μM**, Cayman Chemical, Ann Arbor, MI, Cat. #10004974) **(**Supplementary Materials and Methods).

**Alizarin red staining**

The culture **medium was removed** from each well, and the cells were **washed once with PBS**. Fixation was performed using **1% paraformaldehyde (PFA) in PBS** at **room temperature for 10 min**, followed by **one wash with distilled water**. The cells were then stained with **Alizarin Red staining solution** (PG Research, Tokyo, Japan, Cat. #ARD-A1) at **room temperature for 30 min**. After staining, the solution was removed, and the wells were **washed with distilled water** before imaging. For the **quantification of calcium deposits, calcified nodule dissolution solution** (PG Research, Cat. #ARD-E1) was added to each well and incubated with **gentle agitation for 10 min** to extract the dye. The **absorbance at 450 nm** was then measured using an **iMark Microplate Reader** (Bio-Rad).

**Immunocytochemistry**

Cells were seeded at a density of **1.0 × 10^4^ cells per well** on **BioCoat Collagen Type I eight-well culture slides** (Corning, Cat. #354630) and cultured overnight at **37°C in a 5% CO_2_ incubator**. The medium was then replaced with **DMEM containing 2% FBS** for **4 h of starvation**. After starvation, the medium was switched back to **DMEM containing 10% FBS**, and the following treatments were applied: **BMP2 (100 ng/mL), Yoda1 (10 μM), cyclosporin A (CsA; 100 nM, Fujifilm, Tokyo, Japan, Cat. #031-24931), and** Ionomycin (**1 μM**, Cayman Chemical, Ann Arbor, MI, Cat. #10004974). After **1 h of treatment**, the medium was removed, and the cells were **washed once with PBS**. Fixation was performed using **1% PFA in PBS** at **4°C for 10 min**. The cells were then **washed twice with PBS** and air-dried. Cells were **permeabilized** by incubating with **0.2% Triton X-100** (Sigma Aldrich, Cat. #X100-5 mL) in PBS at **room temperature for 10 min**. Blocking was performed using **1% bovine serum albumin (BSA) in PBS** at **room temperature for 10 min**. After blocking, the cells were incubated overnight at **4°C** with **primary antibodies** diluted in **1% BSA/PBS:** Rabbit Anti-Phospho-SMAD1/5/9 (1:500 dilution; Cell Signaling, Cat. #13820). The next day, the cells were **washed three times with PBS** and then incubated at **room temperature for 30 min** with **secondary antibodies**: Alexa Fluor 488 conjugate (1:500 dilution; Invitrogen, Waltham, MA, Cat. #A-11017) and Alexa Fluor 594 (1:500 dilution; Invitrogen, Cat. #A-11012). After another **three washes with PBS**, fluorescence imaging was performed using an **All-in-One Fluorescence Microscope** (KEYENCE, Osaka, Japan, BZ-X700). For **the quantification of the fluorescence intensity**, the **Fiji application (ImageJ v2.9.0)** was used to measure **the signal intensity** at **five different locations per well**, and the **average intensity** was calculated.

**Immunohistochemistry**

Intervertebral discs from 10-week-old rats were harvested and fixed in 10% formalin for 48 hours. Following fixation, tissues were decalcified in 0.3 M ethylenediaminetetraacetic acid (EDTA; pH 7.5) for 1 week, embedded in paraffin, and sectioned sagittally at a thickness of 4 µm. Sections were baked at 60°C for 30 min, deparaffinized, and rehydrated through a graded ethanol series. Antigen retrieval was performed by heating the slides in 10 mM citrate buffer (pH 6.0) at 121°C for 10 min. After cooling, endogenous peroxidase activity was quenched by incubating the sections in 3% hydrogen peroxide (Fujifilm Wako) for 10 min. The slides were subsequently incubated overnight at 4°C with a primary antibody against PIEZO1 (1:500; Proteintech, Cat. 15939-1-AP). After washing in phosphate-buffered saline (PBS), the sections were incubated with a secondary antibody using the Histofine Simple Stain Rat MAX PO (R) kit (Nichirei Biosciences, Cat. 414181) for 30 min at room temperature. Signal detection was performed using diaminobenzidine (Nichirei Biosciences), with staining intensity monitored under a light microscope. The slides were counterstained with hematoxylin for 30 sec, washed, dehydrated, cleared, and mounted. For each sample, five PIEZO1-immunostained sections were evaluated, and the average number of positive cells per region was quantified.

**Figure legends of the supplementary figures**

**Supplementary Figure S1. Basal expression of mechanoreceptor genes in the AF tissue.**

**(A)** Baseline expression levels of representative mechanoreceptor genes in rat AF cells, assessed by RNA-seq using samples from the CTS-minus (control) group. Multiple mechanoreceptor genes were detectably expressed in rat AF cells, among which *Piezo1* and *Trpv4*—mechanoreceptors previously reported to play functional roles in musculoskeletal tissues—exhibited relatively higher basal expression levels compared with other candidates. **(B)** Gene expression profiling of mechanoreceptor genes in human AF tissue using publicly available microarray data (GEO accession number: GSE70362). Consistent with the rat data, *PIEZO1* and *TRPV4* showed higher expression levels than other mechanoreceptor genes in normal human intervertebral discs, supporting their potential involvement in mechanotransduction in AF tissue.

**Supplementary Figure S2. Intracellular calcium influx in AF cells following Piezo1 activation by Yoda1.**

AF cells were treated with increasing concentrations of Yoda1 (1, 5, 10, 25, and 50 μM), and intracellular calcium influx was measured using a calcium-sensitive fluorescent dye. Calcium influx significantly increased at ≥10 µM, indicating the dose-dependent activation of Piezo1 channels.

**Supplementary Figure S3. Full-length images of the Western blotting shown in Figure 4B and 5C, E.**

Original, uncropped Western blotting images corresponding to the cropped panels in Figure 4B (rat AF cells) , 5C and E (human AF cells) are presented. These images validate the reduction of RUNX2 protein expression after treatment with the Piezo1 agonist Yoda1. **(A, B)**: rat, **(C, D)**: human, Pfirrmann grade 1, **(E, F)**: human, Pfirrmann grade 5

**Supplementary Figure S4. GO analysis of Yoda1-treated AF cells.**

**GO enrichment analysis** revealed that genes downregulated by Piezo1 activation were predominantly associated with cell cycle– and chromosome-related processes, including chromosome organization and DNA replication. In contrast, upregulated genes were enriched in autophagy- and vesicle-related pathways, such as regulation of autophagy and vesicle organization. These findings suggest that Piezo1 activation induces a transcriptional shift from proliferative programs toward intracellular regulatory and signaling processes in AF cells.

**Supplementary Figure S5. Comparison of transcriptomic responses to moderate CTS and pharmacological Piezo1 activation in AF cells.**

**(A)** Venn diagram illustrating the overlap of genes downregulated by moderate CTS and Yoda1 treatment. A total of 135 genes were commonly downregulated under both conditions. Among these, nine genes—including *Runx2*—were annotated to ossification-related GO terms. **(B)** GSEA performed using the ranked gene list demonstrated significant enrichment of the GO term “regulation of ossification” among genes commonly downregulated by CTS and Yoda1 treatment. Together, these data indicate that mechanical stimulation and pharmacological activation of Piezo1 elicit partially overlapping transcriptional programs associated with suppression of ossification-related pathways in AF cells.

**Supplementary Figure S6. Immunocytochemistry following Ionomycin treatment.**

Ionomycin was used as a pharmacological calcineurin activator. Under BMP2 co-treatment, ionomycin reduced nuclear translocation of p-Smad1/5/9, indicating calcineurin-dependent suppression of BMP-Smad signaling.

**Supplementary Figure S7. Immunohistochemistry of rat IVD.**

**(A)** FAST staining of rat IVD. **(B)** Immunohistochemistry for PIEZO1. PIEZO1 was expressed throughout the AF, NP, and CEP, with no appreciable difference in expression levels among these regions.

**Supplementary Figure S8. Bmp2 mRNA expression following Yoda1 treatment.**

Yoda1 treatment tended to increase BMP2 mRNA expression in both rat and human AF cells.

**Supplementary Figure S9. Piezo1 mRNA expression following CTS.**

Piezo1 expression was not significantly altered under either low- and high-intensity CTS.
